# Supplementary material for: Time-dependent catalytic activity in aging condensates
Source: Nat Commun. 2025 Jul 29;16:6959. doi: 10.1038/s41467-025-62074-5 (PMC12307654; doi:10.1038/s41467-025-62074-5)

Supplementary Information

Time-dependent catalytic activity in aging condensates

Wei Kang[1,2,*,†], Zhiyue Wu[1,†], Xinzhi Huang[3,†], Hongbin Qi[1], Jiaxuan Wu[4], Jiahui Wang[1], Jing Li[5], Sijin Wu[4], Byung-Ho Kang[5], Bo Li[3,*], Juncai Ma[5,*], Chuang Xue[1,2,*]

[1]MOE Key Laboratory of Bio-Intelligent Manufacturing, State Key Laboratory of Fine Chemicals, Frontiers Science Centre for Smart Materials Oriented Chemical Engineering, School of Bioengineering, Dalian University of Technology, Dalian 116024, China.

[2]Ningbo Institute of Dalian University of Technology, Ningbo 315016, China.

[3]Department of Mechanical Engineering, Kennesaw State University, Marietta, GA 30060, US.

[4]Jiangsu Province Higher Education Key Laboratory of Cell Therapy Nanoformulation, Wisdom Lake Academy of Pharmacy, Xi'an Jiaotong-Liverpool University, Suzhou 215123, China.

[5]School of Life Sciences, Centre for Cell & Developmental Biology and State Key Laboratory of Agrobiotechnology, The Chinese University of Hong Kong, Shatin, New Territories, Hong Kong, China.

[*]e-mail: kangwei@dlut.edu.cn, bli10@kennesaw.edu, xue.1@dlut.edu.cn, juncaima@cuhk.edu.hk;

[†]These authors contributed equally to this work.

Contents

**Items**	**Page No.**
Supplementary Figure 1. In silica analysis of fibrillarin.	S1
Supplementary Figure 2. SDS-PAGE of purified proteins.	S2
Supplementary Figure 3. Phase diagram of FIBI-GFP-RIAD.	S3
Supplementary Figure 4. Confocal image of 6 μM FIBI-GFP-RIAD in condensate formation buffer.	S4
Supplementary Figure 5. Recruitment of MenH-RIDD into FIB1-GFP-RIAD condensates.	S5
Supplementary Figure 6. Quantitative analysis of fluorescence recovery after photobleaching (FRAP) of the in vitro catalytic condensate.	S6
Supplementary Figure 7. Dilution experiment.	S7
Supplementary Figure 8. Comparison of apparent diffusion coefficients (D_{app}) of probe particles in catalytic condensates.	S8
Supplementary Figure 9. TEM imaging of h0, h4 and h8 biomolecular condensates at low magnification.	S9
Supplementary Figure 10. TEM images of catalytic condensates at h0, h4 and h8 at a series of magnifications.	S10
Supplementary Figure 11. Confocal imaging of biomolecular condensates with or without uranyl acetate (UA) supplementation.	S11
Supplementary Figure 12. All-atom MD simulation results for FIB1-GFP-RIAD/MenH-RIDD modeling system.	S12
Supplementary Figure 13. Comparison of reaction rates among enzymes in sucrose solutions of varying viscosity.	S13
Supplementary Figure 14. Overexpression of FIBI-GFP-RIAD induces the formation of intracellular condensates in living *E. coli*.	S14
Supplementary Figure 15. Efficient recruitment of cargo proteins into intracellular condensates with RIAD-RIDD interaction.	S15
Supplementary Figure 16. Confocal images of Erluc1 at specified time points.	S16
Supplementary Figure 17. Comparison of luciferase expression levels between Erluc0 and Erluc1.	S17

Supplementary Figure 18. Electron microscopy images showing that aged intracellular condensates accumulate protein aggregates.	S18
Supplementary Figure 19. Temporal loss of the pseudo barrier in in vitro catalytic condensates.	S20
Supplementary Figure 20. FRAP analysis of h8 condensates with or without ATP.	S21
Supplementary Figure 21. Comparison of luciferase expression levels between Erluc0 and Erluc1 in the presence of small molecules.	S22
Supplementary Figure 22. Comparison of bacterial growth rates by monitoring OD_{600} with or without small molecules supplementation.	S23
Supplementary Figure 23. Representative confocal images of catalytic condensate over time under different salt concentrations.	S24
Supplementary Figure 24. FRAP analysis of catalytic condensates at h0, h4 and h8 under different NaCl concentrations.	S25
Supplementary Figure 25. Representative confocal images of catalytic condensate over time under different pH values.	S26
Supplementary Figure 26. FRAP analysis of catalytic condensates at h0, h4 and h8 under different pH values.	S27
Supplementary Figure 27. Arginine inhibits condensate aging.	S28
Supplementary Figure 28. Comparison of MenH-catalyzed reaction in catalytic condensates under different salt concentrations.	S29
Supplementary Figure 29. Comparison of relative reaction rates at different pH levels.	S30
Supplementary Figure 30. Calibration curve of SHCHC.	S31
Supplementary Table 1. Plasmids used in this study	S32
Supplementary Table 2. Strains used in this study	S33
Supplementary Table 3. Plasmids construction	S34
Supplementary Table 4. Primers used in this study	S35
Supplementary Table 5. Amino acid sequences of proteins	S36
Supplementary Methods.	S39

Supplementary References.	S43
Uncropped scans of all gels and blots in Supplementary Figures.	S44

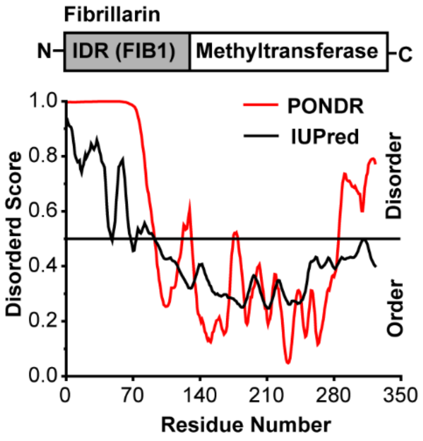

Supplementary Figure 1. In silica analysis of fibrillarin. The N-terminal region of fibrillarin is predicted to be intrinsically disordered using PONDR[1] and IU Pred[2].

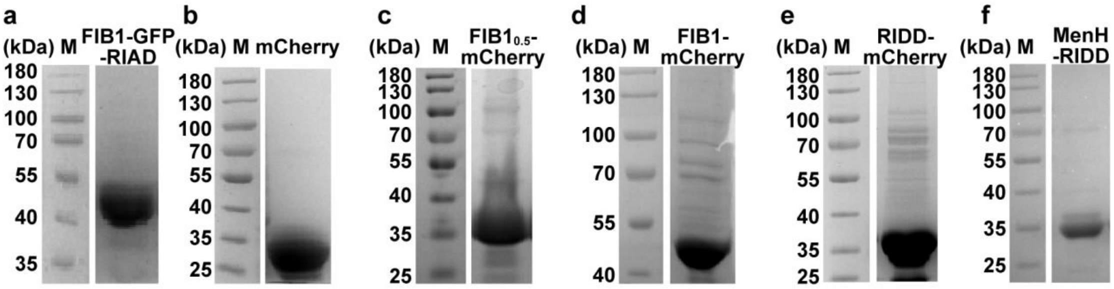

Supplementary Figure 2. SDS-PAGE of purified proteins. a, FIB1-GFP-RIAD (44.39 kDa), **b**, mCherry (28.01 kDa), **c**, FIB1$_{0.5}$-mCherry (35.48 kDa), **d**, FIB1-mCherry (41.93 kDa), **e**, RIDD-mCherry (34.88 kDa), **f**, MenH-RIDD (35.84 kDa). Experiments have been performed with biological replicates at least three times, yielding consistent results. The uncropped scans of all gels are provided at the end of this file.

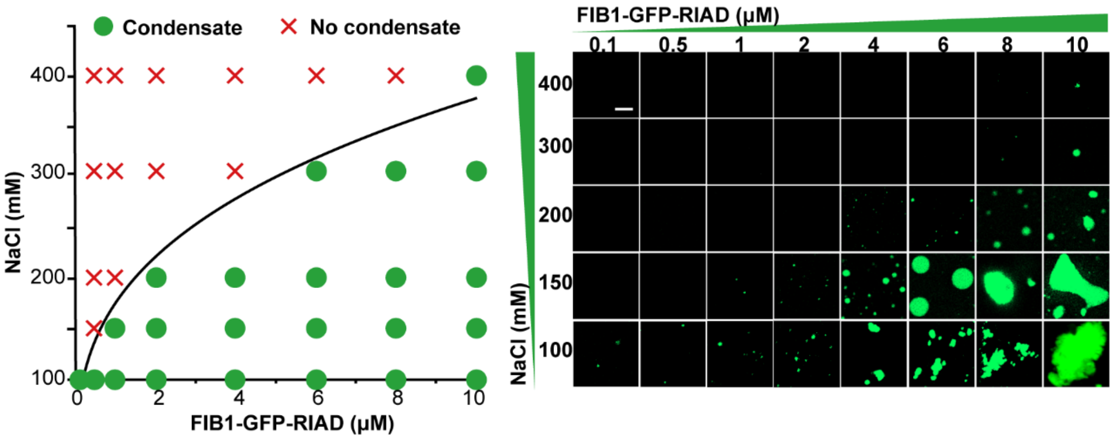

Supplementary Figure 3. Phase diagram of FIBI-GFP-RIAD. Green circles and red crosses donate the presence and the absence of protein condensates, respectively (left). Representative images were acquired under specified concentrations of NaCl and protein (right). Scale bar, 5 μm. Experiments have been performed with biological replicates at least three times with consistent results. Source data are provided as a Source Data file.

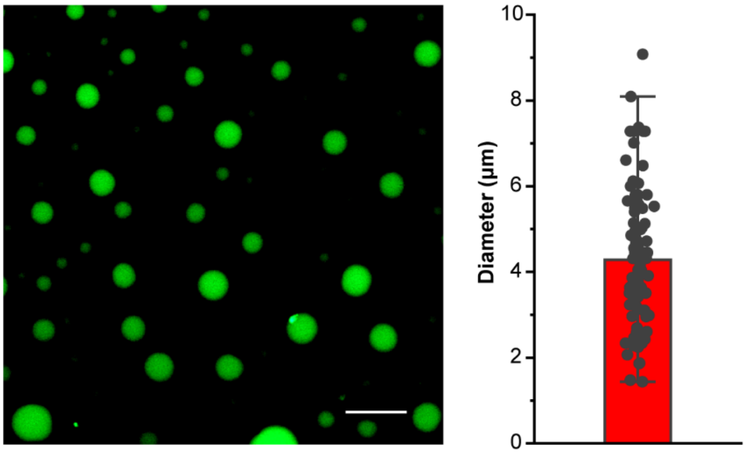

Supplementary Figure 4. Confocal image of 6 µM FIBI-GFP-RIAD in condensate formation buffer. Condensate diameters were quantified using FIJI. A buffer containing 150 mM NaCl, supplemented with 20 mM Tris, 0.5 mM 4-(2-aminoethyl)-benzenesulfonyl fluoride hydrochloride (AEBSF), 0.1 mM dithiothreitol (DTT), 0.1% (v/v) Triton X-100 and 10% (v/v) glycerol, was used as the condensate formation buffer. Data are represented as mean ± SD (n = 100 condensates) from 3 independent experiments. Scale bar, 10 µm. Experiments have been performed with biological replicates at least three times with consistent results. Source data are provided as a Source Data file.

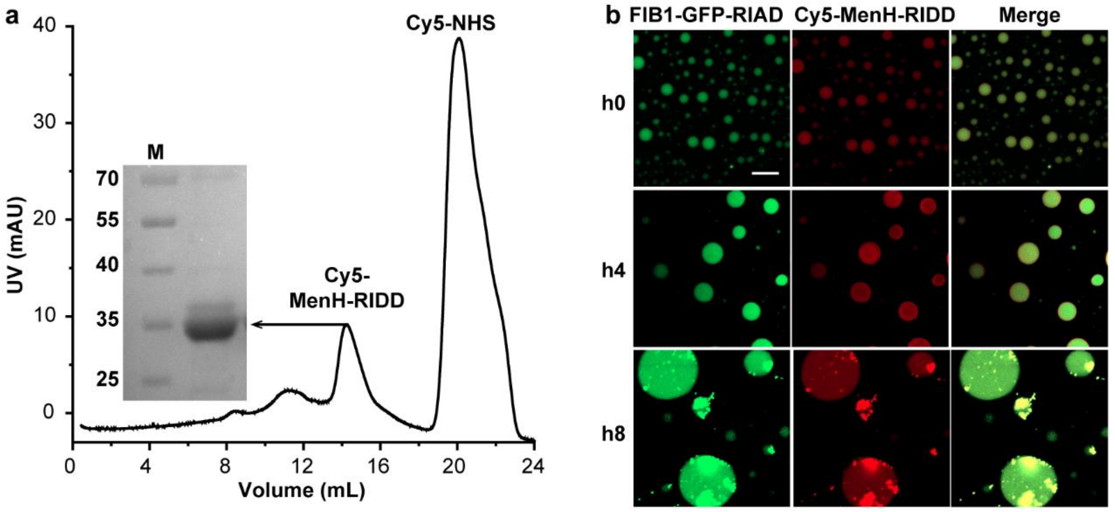

Supplementary Figure 5. Recruitment of MenH-RIDD into FIB1-GFP-RIAD condensates. **a**, Cy5-labeled MenH-RIDD was separated from molar excess Cy5-NHS using size exclusion chromatography (SEC). Cy5-MenH-RIDD was eluted as a single peak at an approximately 13.78 mL. The collected Cy5-MenH-RIDD was subsequently verified by SDS-PAGE. **b**, Representative confocal images of solutions containing 6 μM FIB1-GFP-RIAD and 1 μM MenH-RIDD showing enrichment of enzymes within the condensates. A 1:9 (v/v) ratio of Cy5-labeled to unlabeled MenH-RIDD was used to visualize the enzymes. Scale bar, 10 μm. Experiments have been performed with biological replicates at least three times with consistent results. Source data are provided as a Source Data file.

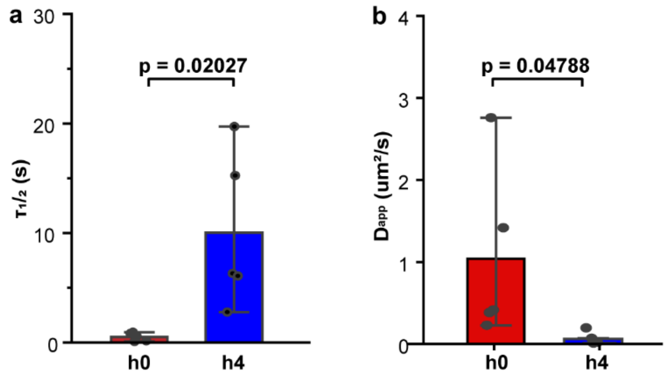

Supplementary Figure 6. Quantitative analysis of fluorescence recovery after photobleaching (FRAP) of the in vitro catalytic condensate. **a,** Half-times of fluorescence recovery ($\tau_{1/2}$). **b**, Apparent diffusion coefficients (D_{app}) calculated from $\tau_{1/2}$. Data are represented as mean ± SD from five independent experiments (n = 5). Source data are provided as a Source Data file.

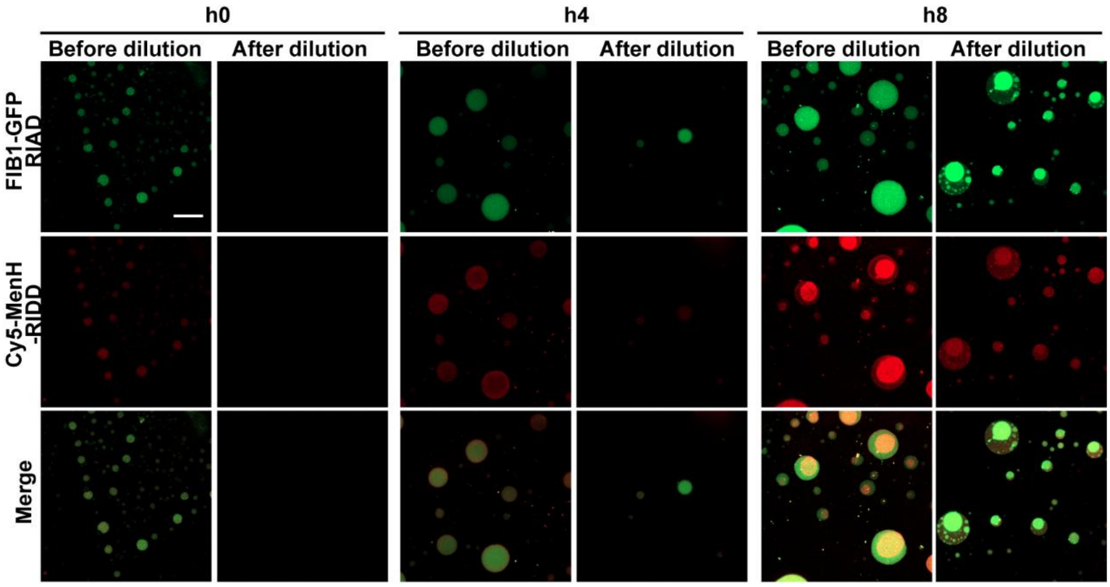

Supplementary Figure 7. Dilution experiment. Representative confocal images of catalytic condensates at different stages before and after dilution are shown. Solutions containing catalytic condensates were diluted at a volume ratio of 1:20 by condensate formation buffer. Condensates at h0 readily dissolve upon dilution, whereas 8-hour-aged condensates remain intact and exhibit resistance to dilution. Scale bar, 10 μm. Experiments have been performed with biological replicates at least three times with consistent results.

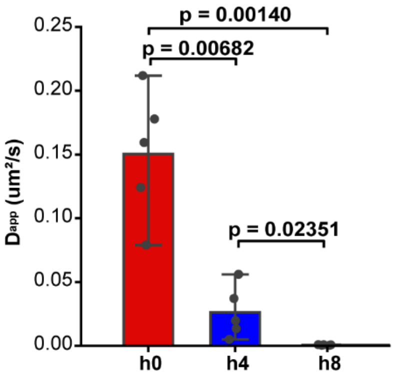

Supplementary Figure 8. Comparison of apparent diffusion coefficients (*D_{app}*) of probe particles in catalytic condensates. D_{app} was determined by particle tracking microrheology (PTM). Data are represented as mean ± SD from five independent experiments (n = 5). Source data are provided as a Source Data file.

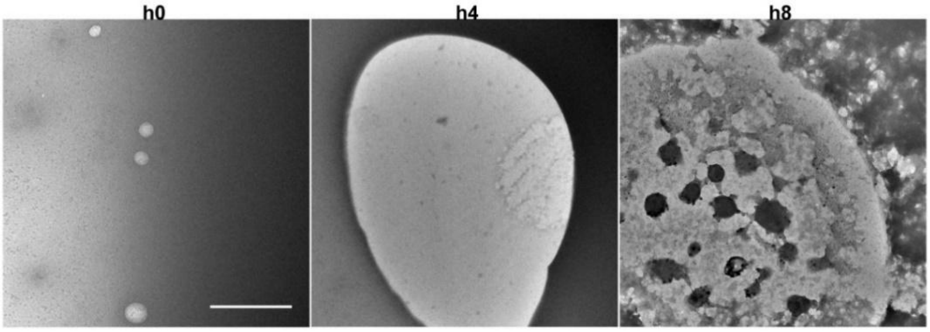

Supplementary Figure 9. TEM imaging of h0, h4 and h8 biomolecular condensates at low magnification. Scale bar, 2 μm. Experiments have been performed with biological replicates at least three times with consistent results.

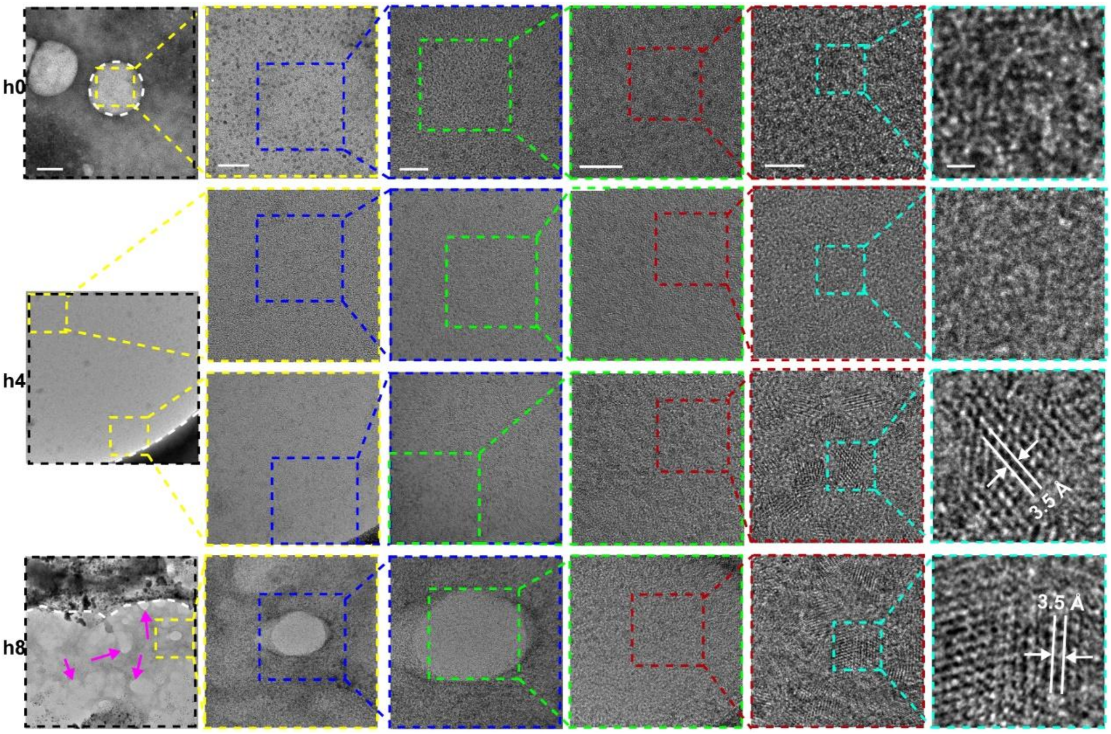

Supplementary Figure 10. TEM images of catalytic condensates at h0, h4 and h8 at a series of magnifications. Colored boxes denote different magnifications. The white dashed lines outline the edges of the condensates. Protein aggregates are marked by magenta arrowheads. Scale bars, 200 nm (black boxes), 50 nm (yellow boxes), 20 nm (blue boxes), 10 nm (green boxes), 5 nm (red boxes) and 1 nm (cyan boxes). Experiments have been performed with biological replicates at least three times with consistent results.

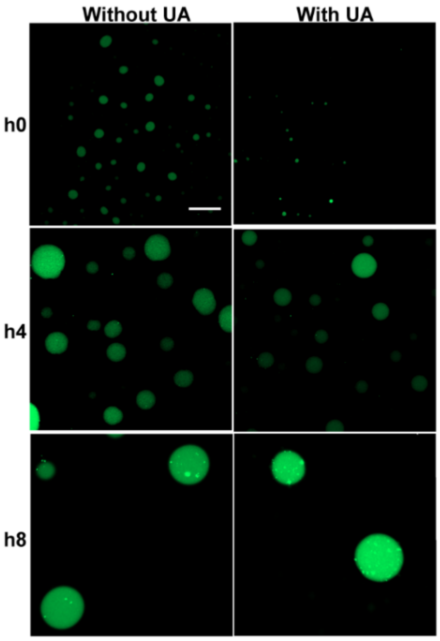

Supplementary Figure 11. Confocal imaging of biomolecular condensates with or without uranyl acetate (UA) supplementation. Representative confocal images of biomolecular condensates at different stages, with or without 2% (w/v) UA treatment, are shown. Condensates at h0 exhibited decreased sizes, whereas those at h4 and h8 showed comparable size after UA treatment, suggesting that the liquid-like biomolecular condensate at h0 are more susceptible to UA. Scale bar, 20 μm. Experiments have been performed with biological replicates at least three times with consistent results.

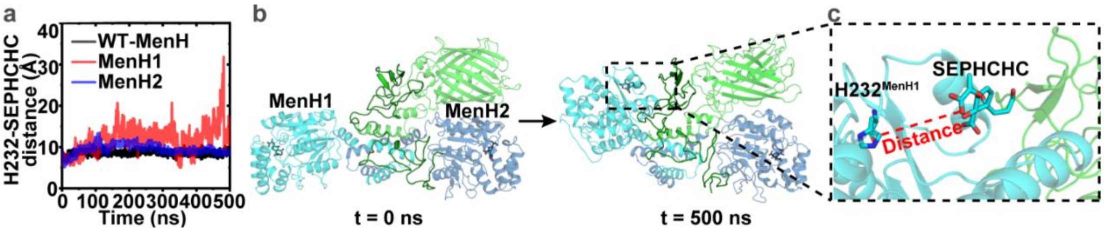

Supplementary Figure 12. All-atom MD simulation results for FIB1-GFP-RIAD/MenH-RIDD modeling system. a, The curves of center of mass distance between His232 and substrate SEPHCHC (2-succinyl-5-enopyruvyl-6-hydroxy-3-cyclohexadiene-1-carboxylate) from different MenH-substrate systems. **b**, Conformational snapshots from MD simulations of the FIB1-GFP-RIAD/MenH-RIDD model system, with MenH1 in cyan, MenH2 in blue, GFP in green, and FIB1 highlighted with a green outline. **c**, A zoomed-in view of the MenH1 active site is shown, with a red dashed line indicating the distance between His232 and the substrate SEPHCHC. Experiments have been performed with biological replicates at least three times with consistent results. Source data are provided as a Source Data file and Supplementary Data 1-6.

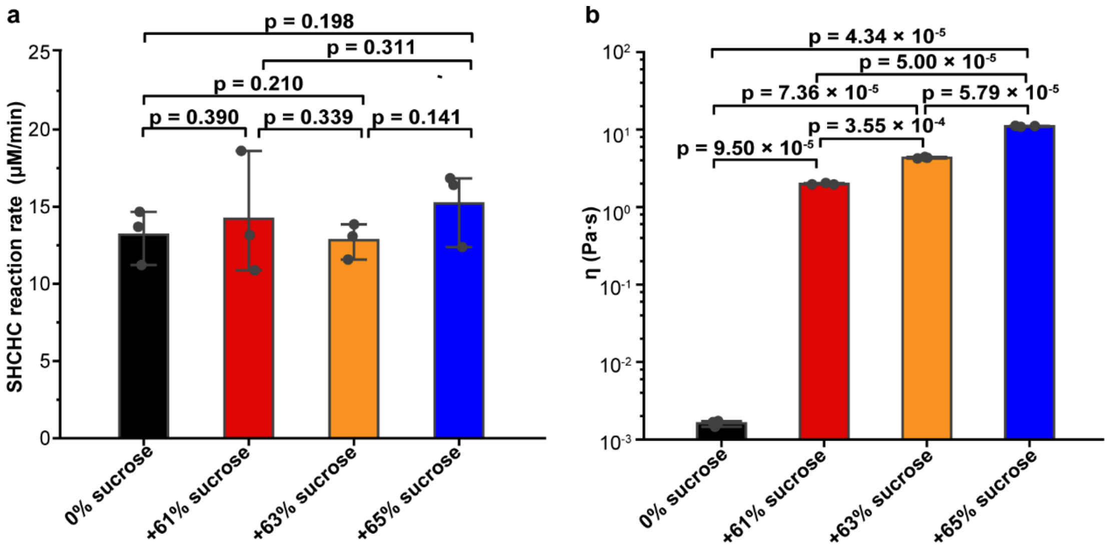

Supplementary Figure 13. Comparison of reaction rates among enzymes in sucrose solutions of varying viscosity. a, Reaction rates of SHCHC production were assessed in sucrose solutions at different concentrations. **b**, Viscosity (η) measurements were conducted on these sucrose solutions using a rheometer. Data are represented as mean ± SD from three independent experiments (n = 3). Source data are provided as a Source Data file.

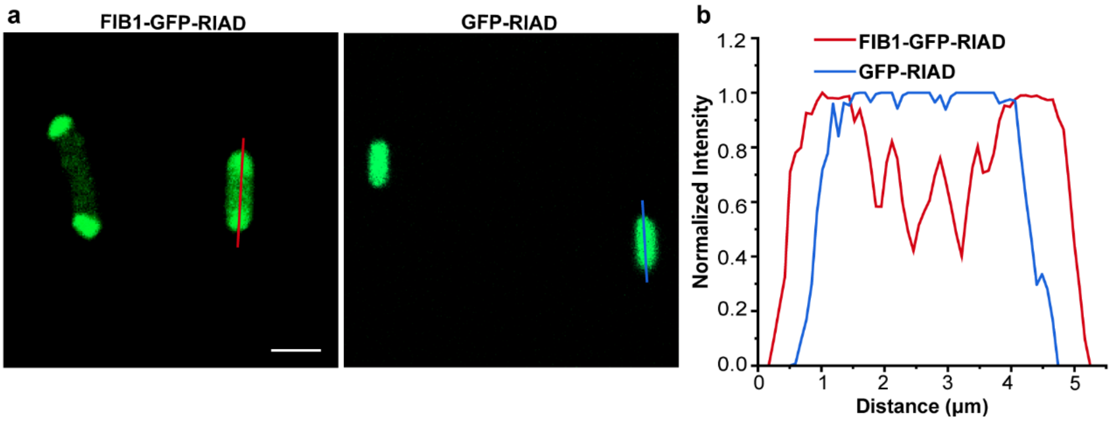

Supplementary Figure 14. Overexpression of FIBI-GFP-RIAD induces the formation of intracellular condensates in living *E. coli*. a, Confocal images of engineered *E. coli* cells expressing either FIBI-GFP-RIAD or GFP-RIAD. Cells expressing FIBI-GFP-RIAD exhibit punctate fluorescence localized at the poles, indicating formation of intracellular condensates (left). In contrast, cells expressing GFP-RIAD alone show a uniform distribution of fluorescence (right). Scale bar, 2 μm. **b**, Quantification of fluorescent intensities along the lines indicated **a**. Experiments have been performed with biological replicates at least three times with consistent results. Source data are provided as a Source Data file.

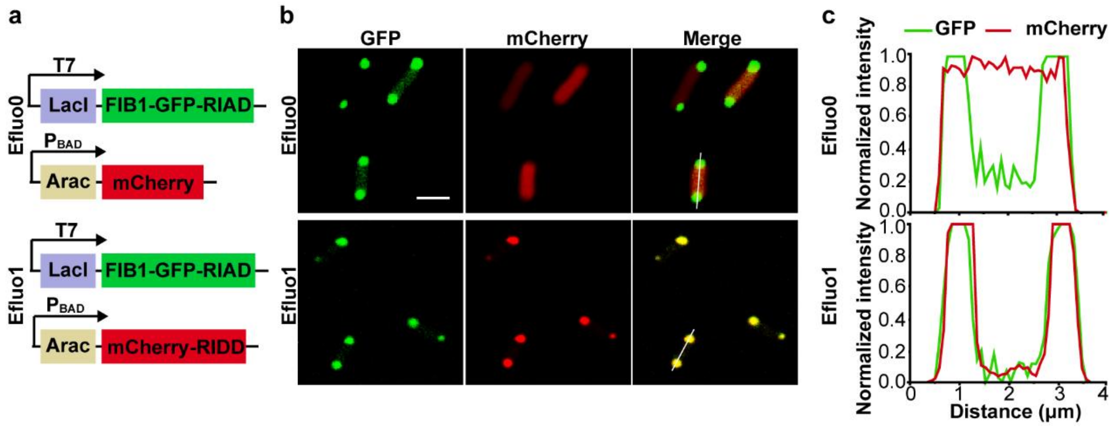

Supplementary Figure 15. Efficient recruitment of cargo proteins into intracellular condensates with RIAD-RIDD interaction. a, Schematic illustration of gene constructs used for the overexpression of FIB1-GFP-RIAD in combination with either mCherry (Efluo0) or mCherry-RIDD (Efluo1). **b**, Representative confocal images of engineered cells. Efluo0 displays a dispersed mCherry signal throughout the cytoplasm, suggesting a lack of specific enrichment of mCherry within the intracellular condensates. Conversely, Efluo1 shows colocalization of the mCherry signal with that of the intracellular condensates, suggesting the specific enrichment of cargo proteins within these condensates. Scale bar, 2 μm. **c**, Normalized fluorescent intensities along the white lines in **b,** showing the co-localization of phase-forming scaffold and mCherry signals. Experiments have been performed with biological replicates at least three times with consistent results. Source data are provided as a Source Data file.

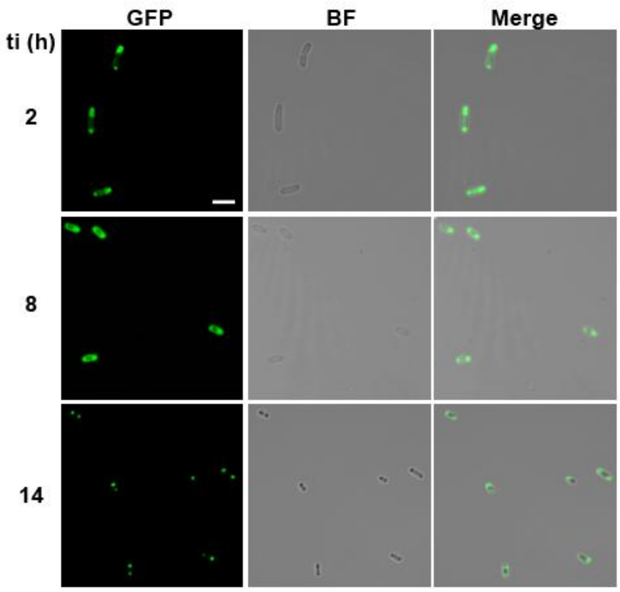

Supplementary Figure 16. Confocal images of Erluc1 at specified time points. The formation of intracellular catalytic condensates is evident, as indicated by the fluorescent puncta localized at the poles of the cells. Erluc1 was imaged at 2, 8 and 14 hours following the induction of FIB1-GFP-RIAD. Scale bar, 5 μm. Experiments have been performed with biological replicates at least three times with consistent results.

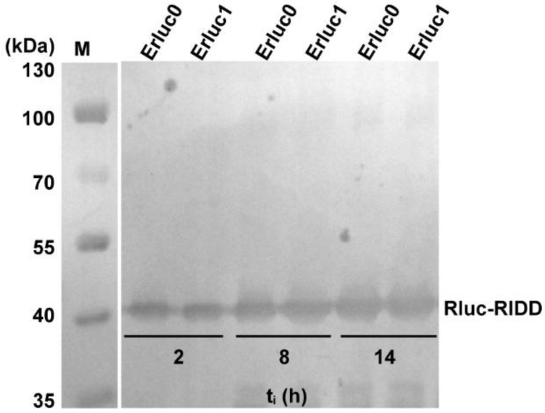

Supplementary Figure 17. Comparison of luciferase expression levels between Erluc0 and Erluc1. Western blot analysis of whole-cell lysates reveals comparable expression levels of Rluc-RIDD (RIDD tagged luciferase) in Erluc0 and Erluc1. These findings indicate that the observed differences in reaction rates are not attributable to variations in protein expression. Experiments have been performed with biological replicates at least three times, yielding consistent results. The uncropped scans of all gels are provided at the end of this file.

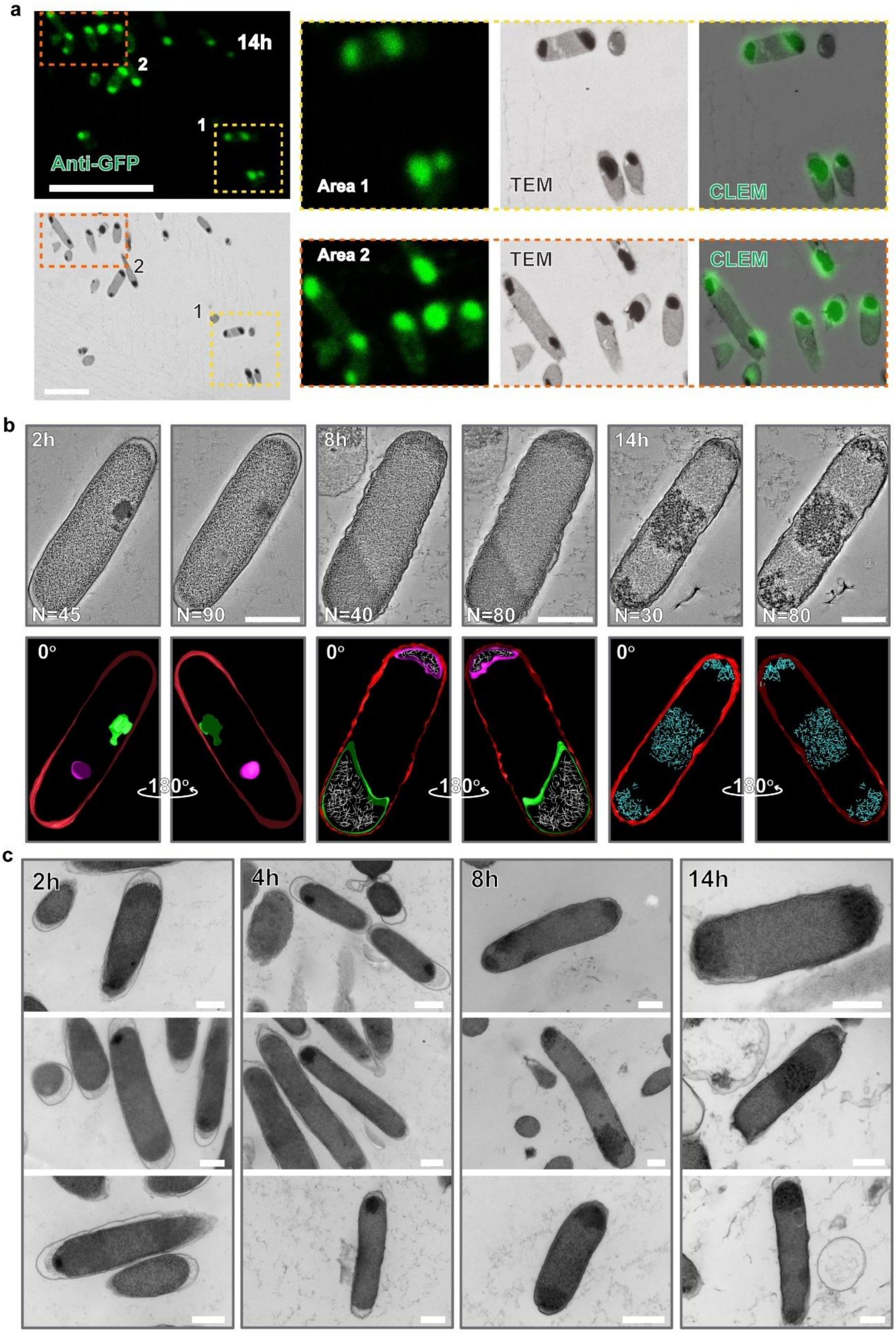

Supplementary Figure 18. Electron microscopy images showing that aged intracellular condensates accumulate protein aggregates. a, Correlative light electron microscopy (CLEM)

of Erluc1 at 14 h. Representative confocal image overview is shown (upper left), with the correlated region further visualized by EM (bottom left). Regions highlighted in yellow and orange boxes are magnified in the right panels. Cells were fixed using high-pressure freezing and embedded in resins. Thin-sections (100 nm) were incubated with an anti-GFP primary antibody followed by a fluorescently tagged secondary antibody. Confocal microscopy images were aligned with TEM images based on cell structures and distribution. Scale bars, 10 μm (confocal) and 4 μm (TEM). **b,** Representative tomographic slice images overview showing the ultrastructure of *E. coli* cells containing intracellular condensates at different stages (top). *E. coli* cells expressing intracellular condensates were fixed and examined using electron tomography. 250 nm thick TEM sections were imaged from multiple angels (± 60°) and aligned together using etomo software. Scale bars, 500 nm. Three-dimensional (3D) models generated from tomographic reconstructions (bottom). Cell membranes are highlighted in red and protein aggregates in white (middle-stage condensates) and blue (late-stage condensates). The colored surfaces around the condensates represent pseudo-barriers between condensates and cytoplasm. **c**, TEM gallery of Erluc1 at 2 h, 4 h, 8 h, and 14 h post-induction. Scale bars, 500 nm. Experiments have been performed with biological replicates at least three times, yielding consistent results. Source data are provided with this paper. Experiments have been performed with biological replicates at least three times with consistent results.

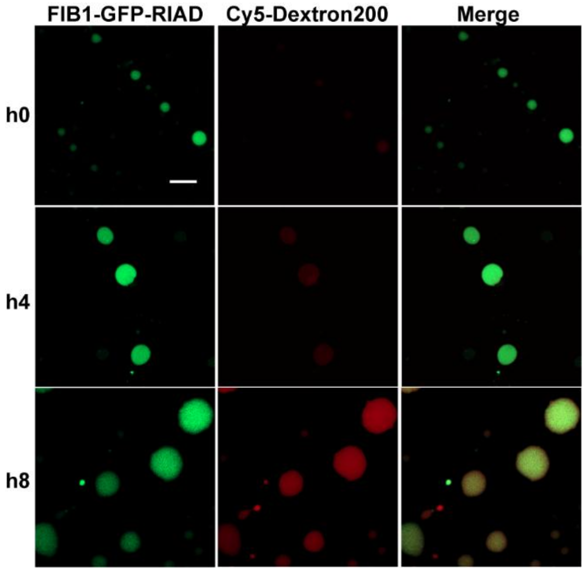

Supplementary Figure 19. Temporal loss of the pseudo barrier in in vitro catalytic condensates. Representative confocal images depict mixtures of catalytic condensates at various stages alongside Cy5-labeled Dextran200 (~ 20 nm, comparable to ribosome size). Scale bar, 10 µm. Experiments have been performed with biological replicates at least three times with consistent results.

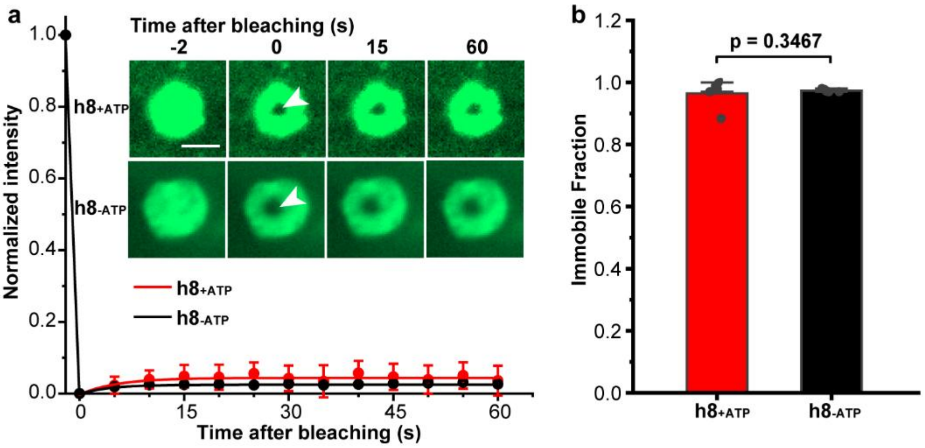

Supplementary Figure 20. FRAP analysis of h8 condensates with or without ATP. a, FRAP recovery curves for 8-hour-aged condensates with ($h8_{+ATP}$) or without ATP ($h8_{-ATP}$). The inset shows representative images of condensates before bleaching, at bleaching, and after bleaching. Scale bar, 2 μm. **b**, Immobile fraction of the condensates derived from **a**. Data are represented as mean ± SD from three independent experiments (n = 3). Source data are provided as a Source Data file.

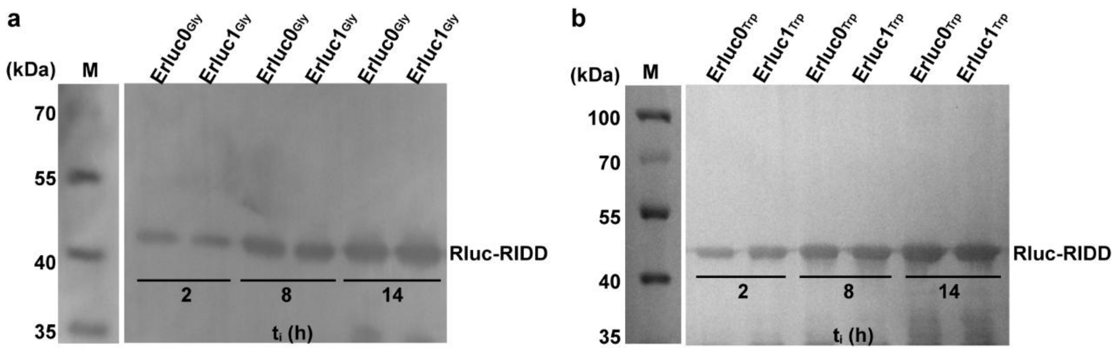

Supplementary Figure 21. Comparison of luciferase expression levels between Erluc0 and Erluc1 in the presence of small molecules. Western blot analysis reveals comparable levels of Rluc-RIDD expression in both Erluc1 and Erluc0 either in the presence of 0.2 mM glycine **a** or 0.2 mM tryptophan **b**. Experiments have been performed with biological replicates at least three times, yielding consistent results. The uncropped scans of all gels are provided at the end of this file.

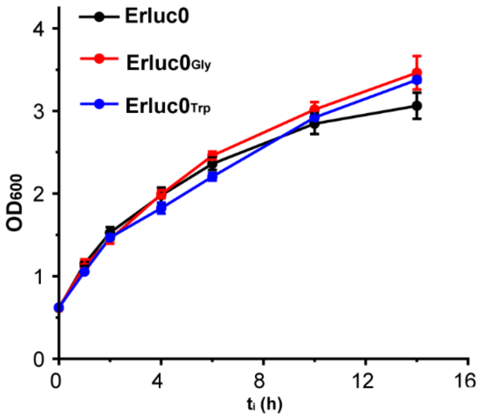

Supplementary Figure 22. Comparison of bacterial growth rates by monitoring OD_{600} with or without small molecules supplementation. The addition of small molecules (0.2 mM glycine or tryptophan) had no significant effect on the growth of engineered strains. Data are represented as mean ± SD from three independent experiments (n = 3). Source data are provided as a Source Data file.

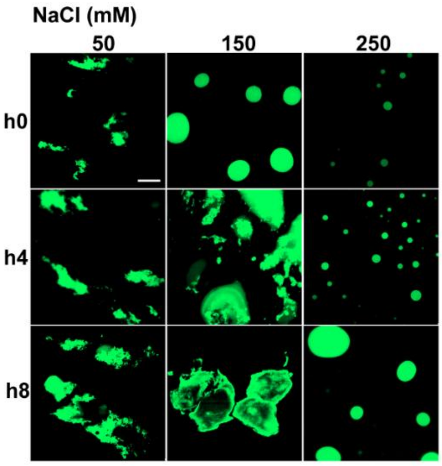

Supplementary Figure 23. Representative confocal images of catalytic condensate over time under different salt concentrations. Solutions containing 10 μM FIB1-GFP-RIAD and 1 μM MenH-RIDD were examined using confocal microscopy. Images were acquired at several time intervals. Scale bar, 20 μm. Experiments have been performed with biological replicates at least three times, yielding consistent results.

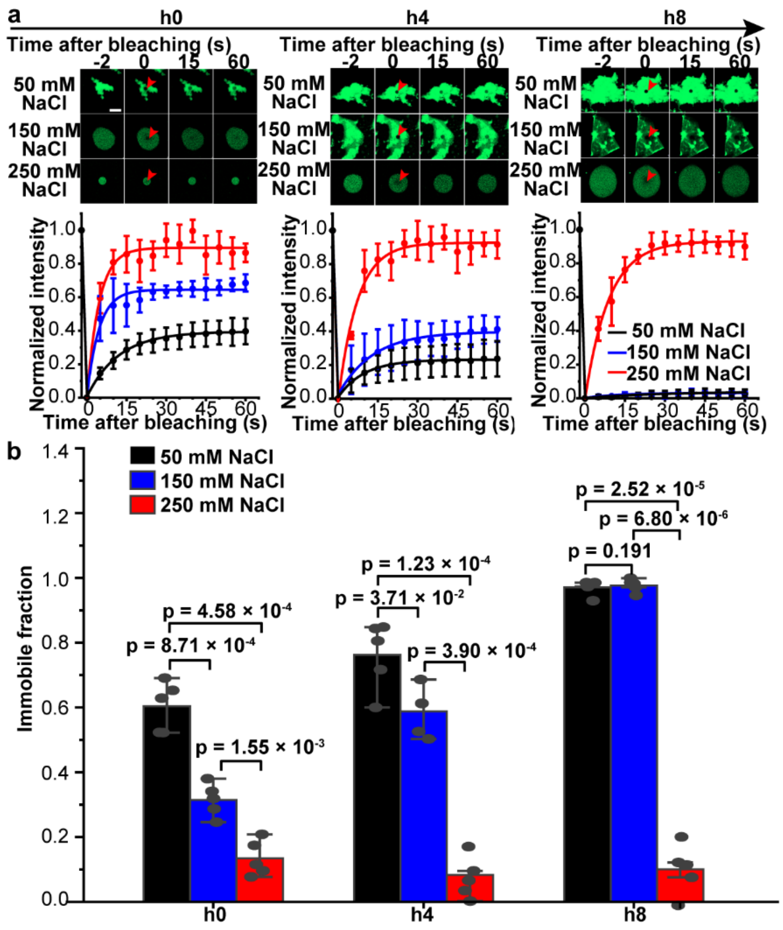

Supplementary Figure 24. FRAP analysis of catalytic condensates at h0, h4 and h8 under different NaCl concentrations. a, Representative images of the condensates before, during, and after bleaching are shown (top). The bleached regions are indicated by red arrows. Scale bar, 10 µm. The FRAP recovery curves of the catalytic condensates are presented (bottom). **b**, Immobile fractions of the condensates derived from **a**. Solutions containing 10 µM FIB1-GFP-RIAD and 1 µM MenH-RIDD were used for FRAP analysis. Data are represented as mean ± SD from three independent experiments (n = 3). Source data are provided as a Source Data file.

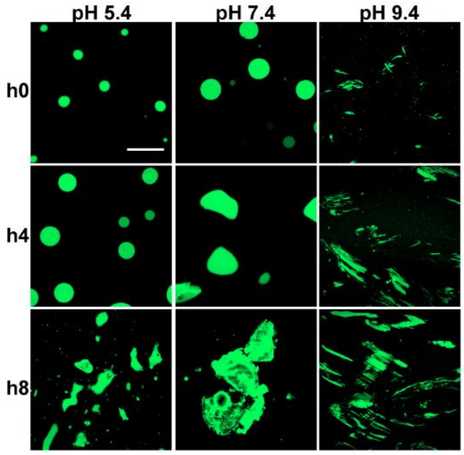

Supplementary Figure 25. Representative confocal images of catalytic condensate over time under different pH values. Solutions containing 10 μM FIB1-GFP-RIAD and 1 μM MenH-RIDD were examined using confocal microscopy. Images were acquired at several time intervals. Scale bar, 20 μm. Experiments have been performed with biological replicates at least three times, yielding consistent results.

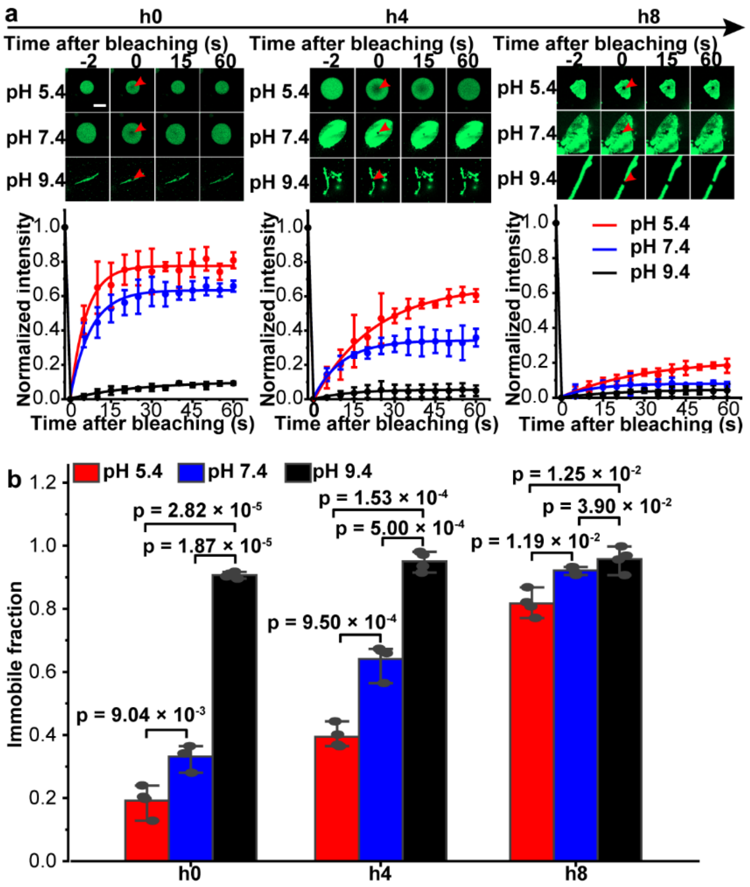

Supplementary Figure 26. FRAP analysis of catalytic condensates at h0, h4 and h8 under different pH values. a, Representative images of the condensates before, during, and after bleaching are shown (top). The bleached regions are indicated by red arrows. Scale bar, 10 μm. The FRAP recovery curves of the catalytic condensates are presented (bottom). **b**, Immobile fractions of the condensates derived from **a**. Solutions containing 10 μM FIB1-GFP-RIAD and 1 μM MenH-RIDD were examined using FRAP. Data are represented as mean ± SD from four independent experiments (n = 4). Source data are provided as a Source Data file.

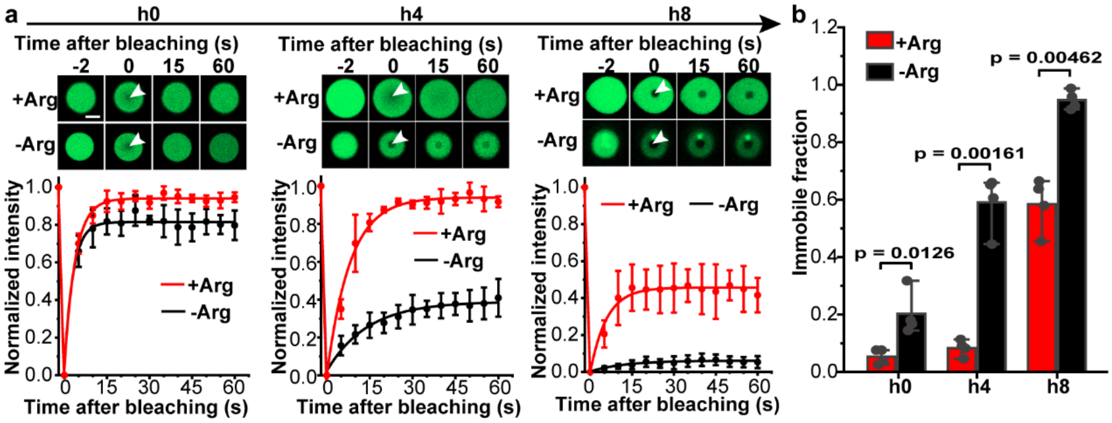

Supplementary Figure 27. Arginine inhibits condensate aging. a, FRAP analysis of biomolecular condensates with (+Arg) or without (-Arg) supplementation of 0.2 mM arginine. Representative images of condensates at various stages following formation, before, at, and after bleaching, are shown (top). The bleached regions are indicated by white arrows. Scale bar, 2 µm. FRAP recovery curves for h0, h4, and h8 condensates are displayed (bottom). Data are expressed as mean ± s.d. (n = 4). **b**, Immobile fractions of condensates with (+Arg) or without (-Arg) arginine at varying ages derived from panel **a.** Data are represented as mean ± SD from four independent experiments (n = 4). Source data are provided as a Source Data file.

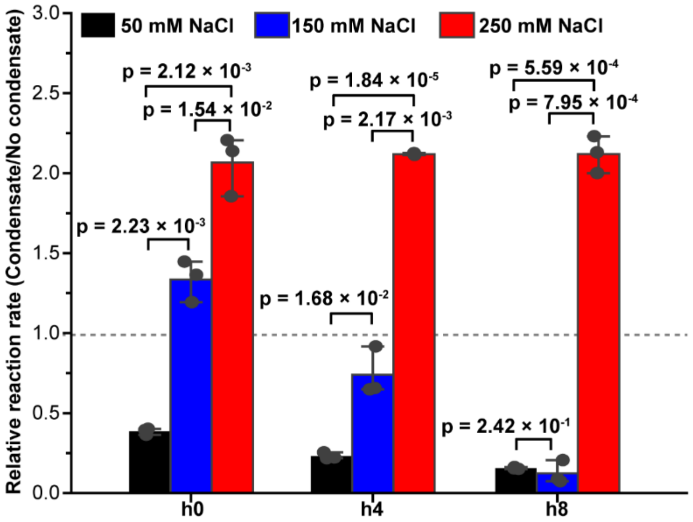

Supplementary Figure 28. Comparison of MenH-catalyzed reaction in catalytic condensates under different salt concentrations. Reaction rates for the catalytic condensates were normalized against a control system containing identical enzyme and NaCl concentration but lacking condensates. The gray dashed line indicates a relative reaction rate of 1, representing a comparable rate between the system containing condensates and the control system. Data are represented as mean ± SD from three independent experiments (n = 3). Source data are provided as a Source Data file.

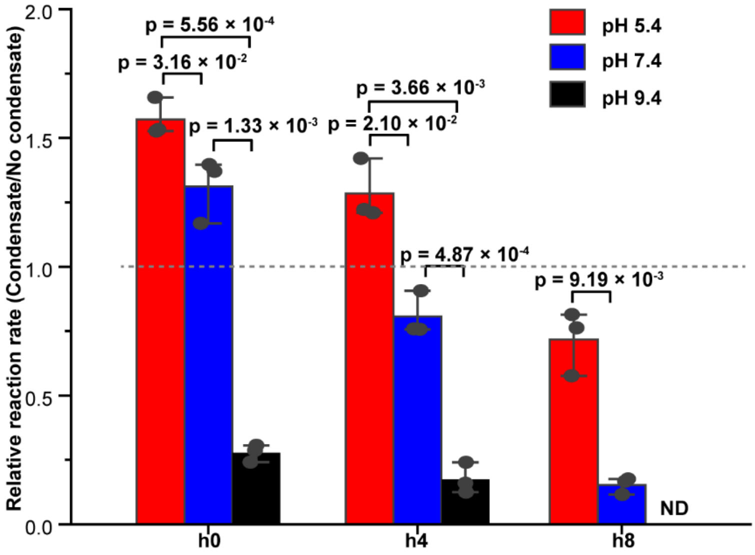

Supplementary Figure 29. Comparison of relative reaction rates at different pH levels. Reaction rates for condensates were normalized to a control system containing identical enzyme and pH values but lacking condensates. ND (not detected): SHCHC absorbance signals were undetectable in h8 systems at pH 9.4, regardless of condensate presence. The gray dashed line indicates a relative reaction rate of 1, representing a comparable rate between the system containing condensates and the control system. Data are represented as mean ± SD from three independent experiments (n = 3). Source data are provided as a Source Data file.

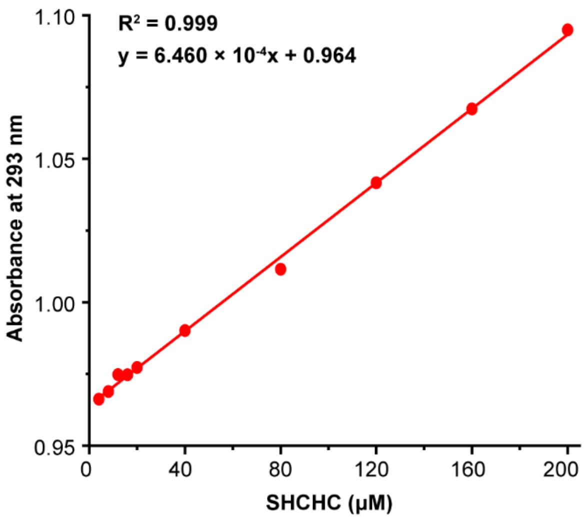

Supplementary Figure 30. Calibration curve of SHCHC. The concentration of the product SHCHC, and its specific absorbance at 293 nm were linearly fitted as a standard curve with an R^2 value > 0.99. Source data are provided as a Source Data file.

Supplementary Table 1. Plasmids used in this study

Plasmids	Backbone	Description	Source
pET28a- FIB1-GFP-RIAD	pET28a (+)	N-terminal His-tagged FIB1-GFP, linker and RIAD, Kan^+	this study
pET28a-mCherry	pET28a (+)	N-terminal His-tagged mCherry, Kan^+	laboratory stock
pET28a- $FIB1_{0.5}$-mCherry	pET28a (+)	N-terminal His-tagged $FIB1_{0.5}$, linker and mCherry, Kan^+	this study
pET28a- FIB1-mCherry	pET28a (+)	N-terminal His-tagged FIB1, linker and mCherry, Kan^+	this study
pET28a-RIDD-mCherry	pET28a (+)	N-terminal His-tagged RIDD, linker and mCherry, Kan^+	this study
pET28a-MenH-RIDD	pET28a (+)	N-terminal His-tagged MenH, linker and RIDD, Kan^+	this study
pACYC- GFP-RIAD	pACYC-Duet1	N-terminal His-tagged GFP, linker and RIAD, Cm^+	this study
pACYC- FIB1-GFP-RIAD	pACYC-Duet1	N-terminal His-tagged FIB1-GFP, linker and RIAD, Cm^+	this study
pBAD-mCherry	pBAD-HisA	N-terminal His-tagged mCherry, Amp^+	this study
pBAD -mCherry-RIDD	pBAD-HisA	N-terminal His-tagged mCherry, linker and RIDD, Amp^+	this study
pBAD-mCherry-ELK16	pBAD-HisA	N-terminal His-tagged mCherry-ELK16, Amp^+	this study
pBAD-Rluc-RIDD	pBAD-HisA	N-terminal S-tagged Rluc, linker and RIDD, Amp^+	this study

Supplementary Table 2. Strains used in this study

Strains	Description	Source
Efluo0	*E. coli* BL21 (DE3) with pACYC-FIB1-GFP-RIAD and pBAD-mCherry	this study
Efluo1	*E. coli* BL21 (DE3) with pACYC-FIB1-GFP-RIAD and pBAD-mCherry-RIDD	this study
Erluc0	*E. coli* BL21 (DE3) with pACYC-Duet1 and pBAD-Rluc-RIDD	this study
Erluc1	*E. coli* BL21 (DE3) with pACYC-FIB1-GFP-RIAD and pBAD-Rluc-RIDD	this study

Supplementary Table 3. Plasmids construction

Plasmids	Fragments	Template	Primers
pET28a- $FIB1_{0.5}$-mCherry	$FIB1_{0.5}$	pET28a- FIB1-GFP-RIAD	FIB1-F<br>0.5FIB1-R
	pET28a-mCherry	pET28a-mCherry	pm-F<br>p28-R
pET28a- FIB1-mCherry	FIB1	pET28a- FIB1-GFP-RIAD	FIB1-F<br>FIB1-R
	pET28a-mCherry	pET28a-mCherry	pmCh-F<br>p28-R
pET28a-RIDD-mCherry	mCherry	pET28a-mCherry	mCherry-F<br>mCherry-R
	pET28a-RIDD	pET28a-RIDD	pRIDD-F<br>pRIDD-R
pET28a-MenH-RIDD	MenH	*E. coli*	MenH-F<br>MenH-R
	pET28a-RIDD	pET28a-RIDD	pMenH-F<br>pMenH-R
pACYC-GFP-RIAD	GFP-RIAD	pET28a- FIB1-GFP-RIAD	GFP-F<br>RIAD-R
	pACYC-Duet1	pACYCDuet1	pACYC-RIAD-F<br>pACYC-GFP-R
pACYC-FIB1-GFP-RIAD	FIB1-GFP-RIAD	pET28a- FIB1-GFP-RIAD	FIB1-F<br>RIAD-R
	pACYC-Duet1	pACYCDuet1	pACYC-RIAD-F<br>pACYC-FIB1-R
pBAD-mCherry	mCherry	pET28a-mCherry	his-mCh-F<br>mCherry-R
	pBAD	pBAD-Rluc-RIDD	pBAD-F<br>pBAD-R
pBAD-mCherry-RIDD	mCherry	pET28a-mCherry	his-mCh-F<br>mCherry-R
	pBAD-RIDD	pBAD-Rluc-RIDD	pBAD-RIDD-F<br>pBAD-R
pBAD-mCherry-ELK16	mCherry	pET28a-mCherry	his-mCh -F<br>ELK-R
	pBAD	pBAD-Rluc-RIDD	pELK-F<br>pBAD-R

Supplementary Table 4. Primers used in this study

Primers	Sequence 5’-3’ (restriction sites are underlined)
FIB1-F	CGCCCTGGTTTTAGCC
0.5FIB1-R	ACCCGGACTGCTGAAACCGC
pm-F	CGGTTTCAGCAGTCCGGGTGGTGGTGGTGGTTCAGTGA GCAA
p28-R	GGGCTAAAACCAGGGCGGCCGCTGCTGTGATGATGAT
FIB1-R	AATACGTTTTTCACCATACACGCTTTCG
pmCh-F	TGTATGGTGAAAAACGTATTGGTGGTGGTGGTTCAGTG AGCAAGGGCGAGGAG
mCherry-F	GTGAGCAAGGGCGAGGAGG
mCherry-R	CTTGTACAGCTCGTCCATGCC
pRIDD-F	GCATGGACGAGCTGTACAAGTAACGCGGATCCGAATTC GAG
pRIDD-R	CCTCCTCGCCCTTGCTCACTGAACCACCACCACCTTTGG CTTCTTCTTTTTCCAGACGT
MenH-F	ATCCTGCACGCGCAGGCAAA
MenH-R	GAAACGCAAGATCTGCGCCAGAC
pMenH-F	GTCTGGCGCAGATCTTGCGTTTCGGTACCGGTGGTGGT GGTTC
pMenH-R	TTTGCCTGCGCGTGCAGGATGCCGCTGCTGTGATGATG AT
GFP-F	ATGAGTAAAGGCGAAGAACTGTTTACCG
RIAD-R	GCAACCTTCGGTAGCTTCTTTAATGA
pACYC-RIAD-F	AAGCTACCGAAGGTTGCTAAGAATTCGAGCTCGGCGC
pACYC-GFP-R	AGTTCTTCGCCTTTACTCATTGGATCCTGGCTGTGGTGA T
pACYC-FIB1-R	CGCGGGCTGAAACCCGGACGGCCGCTGCTGTGGTGAT
his-mCh-F	AGCAGCCATCATCATCATCATCACAG
pBAD-F	GGCATGGACGAGCTGTACAAGTAAGGTACCATATGGGA ATT
pBAD-R	GATGATGATGATGGCTGCTTCCCATGGTTAATTCCTCCT GTTAGC
pBAD-RIDD-F	CATGGACGAGCTGTACAAGGGTACCGGTGGTGGTGGTT
ELK-R	GTTCCAGTTCCAGTTTCAGTTTCAGTTCCAGTTCCAGCT TGTACAGCTCGTCCATGC
pELK-F	ACTGAAACTGGAACTGGAACTGAAACTGAAATAAGGT ACCATATGGGAATTCGAAGCT

Supplementary Table 5. Amino acid sequences of the proteins

Proteins	Amino Acids Sequence
FIB1-GFP-RIAD	MGSSHHHHHHSSGRPGFSPRGCRGGFGDRGGFGGRG GFGDRGGFRGGSRGGFGGRGRGGDRGGRGGFRGGFS SPGRGGPRGGGRGGFGGGRGGFGAGRKVIVEPHRHE GIFICRGKEDALVTKNLVPGESVYGEKRIGGGSSKGEE LFTGVVPILVELDGDVNGHKFSVRGEGEGDATNGKLT LKFICTTGKLPVPWPTLVTTLTYGVQCFSRYPDHMKR HDFFKSAMPEGYVQERTISFKDDGTYKTRAEVKFEGD TLVNRIELKGIDFKEDGNILGHKLEYNFNSHNVYITAD KQKNGIKANFKIRHNVEDGSVQLADHYQQNTPIGDGP VLLPDNHYLSTQSVLSKDPNEKRDHMVLLEFVTAAGI THGMDELYKKLGSGGGGSGGGGCGLEQYANQLADQ IIKEATE*
mCherry	MGSSHHHHHHSSGVSKGEEDNMAIIKEFMRFKVHME GSVNGHEFEIEGEGEGRPYEGTQTAKLKVTKGGPLPF AWDILSPQFMYGSKAYVKHPADIPDYLKLSFPEGFKW ERVMNFEDGGVVTVTQDSSLQDGEFIYKVKLRGTNFP SDGPVMQKKTMGWEASSERMYPEDGALKGEIKQRL KLKDGGHYDAEVKTTYKAKKPVQLPGAYNVNIKLDI TSHNEDYTIVEQYERAEGRHSTGGMDELYKLEHHHH HH*
FIB1$_{0.5}$-mCherry	MGSSHHHHHHSSGRPGFSPRGGRGGFGDRGGFGGRG GFGDRGGFRGGSRGGFGGRGRGGDRGGRGGFRGGFS SPGGGGGSVSKGEEDNMAIIKEFMRFKVHMEGSVNG HEFEIEGEGEGRPYEGTQTAKLKVTKGGPLPFAWDILS PQFMYGSKAYVKHPADIPDYLKLSFPEGFKWERVMN FEDGGVVTVTQDSSLQDGEFIYKVKLRGTNFPSDGPV MQKKTMGWEASSERMYPEDGALKGEIKQRLKLKDG GHYDAEVKTTYKAKKPVQLPGAYNVNIKLDITSHNE DYTIVEQYERAEGRHSTGGMDELYKLEHHHHHH*
FIB1-mCherry	MGSSHHHHHHSSGRPGFSPRGGRGGFGDRGGFGGRG GFGDRGGFRGGSRGGFGGRGRGGDRGGRGGFRGGFS SPGRGGPRGGGRGGFGGGRGGFGAGRKVIVEPHRHE GIFICRGKEDALVTKNLVPGESVYGEKRIGGGGSVSK GEEDNMAIIKEFMRFKVHMEGSVNGHEFEIEGEGEGR PYEGTQTAKLKVTKGGPLPFAWDILSPQFMYGSKAY VKHPADIPDYLKLSFPEGFKWERVMNFEDGGVVTVT QDSSLQDGEFIYKVKLRGTNFPSDGPVMQKKTMGWE ASSERMYPEDGALKGEIKQRLKLKDGGHYDAEVKTT YKAKKPVQLPGAYNVNIKLDITSHNEDYTIVEQYERA EGRHSTGGMDELYKLEHHHHHH*
RIDD-mCherry	MGSSHHHHHHSSGLRECELYVQKHNIQALLKDSIVQL CTARPERPMAFLREYFERLEKEEAKGGGGSVSKGEED

	NMAIIKEFMRFKVHMEGSVNGHEFEIEGEGEGRPYEG TQTAKLKVTKGGPLPFAWDILSPQFMYGSKAYVKHP ADIPDYLKLSFPEGFKWERVMNFEDGGVVTVTQDSSL QDGEFIYKVKLRGTNFPSDGPVMQKKTMGWEASSER MYPEDGALKGEIKQRLKLKDGGHYDAEVKTTYKAK KPVQLPGAYNVNIKLDITSHNEDYTIVEQYERAEGRH STGGMDELYKLEHHHHHH*
MenH-RIDD	MGSSHHHHHHSSGILHAQAKHGKPGLPWLVFLHGFS GDCHEWQEVGEAFADYSRLYVDLPGHGGSAAISVDG FDDVTDLLRKTLVSYNILDFWLVGYSLGGRVAMMAA CQGLAGLCGVIVEGGHPGLQNAEQRAERQRSDRQWV QRFLTEPLTAVFADWYQQPVFASLNDDQRRELVALR SNNNGATLAAMLEATSLAVQPDLRANLSARTFAFYY LCGERDSKFRALAAELAADCHVIPRAGHNAHRENPA GVIASLAQILRFGTGGGGSGGGGCGSLRECELYVQKH NIQALLKDSIVQLCTARPERPMAFLREYFERLEKEEAK *
GFP-RIAD	MGSSHHHHHHSSGSKGEELFTGVVPILVELDGDVNG HKFSVRGEGEGDATNGKLTLKFICTTGKLPVPWPTLV TTLTYGVQCFSRYPDHMKRHDFFKSAMPEGYVQERTI SFKDDGTYKTRAEVKFEGDTLVNRIELKGIDFKEDGNI LGHKLEYNFNSHNVYITADKQKNGIKANFKIRHNVED GSVQLADHYQQNTPIGDGPVLLPDNHYLSTQSVLSKD PNEKRDHMVLLEFVTAAGITHGMDELYKKLGSGGGG SGGGGCGLEQYANQLADQIIKEATE*
mCherry-RIDD	MGSSHHHHHHSSGVSKGEEDNMAIIKEFMRFKVHME GSVNGHEFEIEGEGEGRPYEGTQTAKLKVTKGGPLPF AWDILSPQFMYGSKAYVKHPADIPDYLKLSFPEGFKW ERVMNFEDGGVVTVTQDSSLQDGEFIYKVKLRGTNFP SDGPVMQKKTMGWEASSERMYPEDGALKGEIKQRL KLKDGGHYDAEVKTTYKAKKPVQLPGAYNVNIKLDI TSHNEDYTIVEQYERAEGRHSTGGMDELYKGTGGGG SGGGGCGSLRECELYVQKHNIQALLKDSIVQLCTARP ERPMAFLREYFERLEKEEAK*
Rluc-RIDD	MGKETAAAKFERQHMDSASKVYDPEQRKRMITGPQ WWARCKQMNVLDSFINYYDSEKHAENAVIFLHGNAT SSYLWRHVVPHIEPVARCIIPDLIGMGKSGKSGNGSYR LLDHYKYLTAWFELLNLPKKIIFVGHDWGSALAFHY AYEHQDRIKAIVHMESVVDVIESWKNWPDIEEELALI KSEEGEKMVLENNFFVETVLPSVIMRKLEPEEFAAYL EPFKEKGEVRRPTLSWPREIPLVKGGKPDVVQIVRNY NAYLRASDDLPKLFIESDPGWFSNAIIEGAKKFPNTEF VKVKGLHFLQEDAPDEMGKYIKSFVERVLKNEQLEG

	GGGSGGGGCGSLRECELYVQKHNIQALLKDSIVQLCT ARPERPMAFLREYFERLEKEEAK*
mCherry-ELK16	MGSSHHHHHHSSGVSKGEEDNMAIIKEFMRFKVHME GSVNGHEFEIEGEGEGRPYEGTQTAKLKVTKGGPLPF AWDILSPQFMYGSKAYVKHPADIPDYLKLSFPEGFKW ERVMNFEDGGVVTVTQDSSLQDGEFIYKVKLRGTNFP SDGPVMQKKTMGWEASSERMYPEDGALKGEIKQRL KLKDGGHYDAEVKTTYKAKKPVQLPGAYNVNIKLDI TSHNEDYTIVEQYERAEGRHSTGGMDELYKLELELKL KLELELKLK*

Supplementary Methods

Chemicals and reagents. PCR (Polymerase chain reaction) primers used in this study were obtained from Sangon Biotech. The polymerase used for the PCR reaction included Phanta Super-Fidelity DNA Polymerase (Vazyme, Cat. No.: P501-d3) or Taq DNA polymerase (TaKaRa, Cat. No.: R001A), selected according to the fidelity requirements. Purification of PCR fragments and plasmid extraction were performed using a gel extraction kit (Cat. No.: D2500-03) and a plasmid extraction kit (Cat. No.: D6945-02), respectively, from Omega Bio-Tek. Restriction enzymes for cloning were bought from NEB. Gibson assembly was conducted with a kit from Vazyme (Cat. No.: C115-01). Lysogeny broth (LB) was acquired from Invitrogen (Cat. No.: 12780052), while antibiotics were sourced from BBI. Chorismate was a product of Sigma (Cat. No.: 617-12-9) and coelenterazine was obtained from MedChemExpress (MCE, Cat. No.: HY-18743). Glycine (Cat. No.: A110752), tryptophan (Cat. No.: L769392) and adenosine triphosphate (ATP, Cat. No.: HY-B2176R) were acquired from Aladdin, Macklin and MCE, respectively.

Protein expression and purification. The protein expression vector containing the gene of interest was transformed into homemade *E. coli* BL21(DE3) competent cells, which were subsequently grown on agar plates supplemented with the appropriate antibiotic at 37°C. A positive colony was selected and inoculated into 10 mL LB media for overnight growth at 37°C. The overnight cell culture was then transferred into 800 mL LB at a 1:100 dilution, also supplemented with the same antibiotic. Cells were incubated at 37 °C with shaking at approximately 200 rpm. Protein expression was induced when the optical density of the cell culture at 600 nm (OD_{600}) reached ~0.6 by adding isopropyl D-thiogalactopyranoside (IPTG) to a final concentration of 1 mM. For proteins without IDR, cells continued to grow for an additional 16 hours at 16 °C following IPTG induction. In contrast, proteins containing IDRs were expressed for a shorter duration of 6 hours post IPTG addition to minimize the formation of insoluble aggregates due to condensate aging.

To purify proteins containing IDRs and avoid unintended phase separation, cell pellets were resuspended in 50 mL of binding buffer with high NaCl concentrations at pH 7.4. The binding buffer contained 20 mM Tris, 500 mM NaCl, 0.5 mM 4-(2-aminoethyl)-benzenesulfonyl fluoride hydrochloride (AEBSF), 0.1 mM dithiothreitol (DTT), 0.1% (v/v) Triton X-100, and 10% (v/v) glycerol. Cells were lysed using a high-pressure homogenizer (Atshph) for 15 minutes. The lysate was then centrifuged at 13,000 × g for 20 minutes at 4 °C. The supernatant was filtered through a 0.22 μm membrane and loaded onto a HisTrap HP column (Cytiva) which had been pre-equilibrated with the same binding buffer. Recombinant proteins were purified using an AKTA pure fast protein liquid chromatography (FPLC, Cytiva) system equipped with the HisTrap HP column (Cytiva). The target protein was eluted using a gradient of elution buffer (20 mM Tris, 500 mM NaCl, 500 mM imidazole, 0.5 mM AEBSF, 0.1 mM DTT, 0.1% Triton X-100, 10% glycerol, pH 7.8), increasing linearly from 0% to 100% over 50 mL. Desalting of the purified protein was performed using FPLC with a HiTrap desalting column (Cytiva). The purified proteins were aliquoted and then stored at -80 °C in a stock buffer composed of high NaCl concentration (20 mM Tris, 500 mM NaCl, 0.5 mM AEBSF, 0.1 mM DTT, 0.1% Triton X-100, 10% glycerol, pH 7.4). Protein concentration was

determined using the BCA methods.

For enzyme purification, the procedure was identical to that described above, with the exception that the NaCl concentration in the binding, elution, and stock buffers was set to 150 mM.

Phase diagram. To construct the phase diagram of FIB1-IDR-RIAD, 20 μL solutions containing varying concentrations of NaCl and protein were prepared. A no-salt buffer consisting of 20 mM Tris, 0.5 mM AEBSF, 0.1 mM DTT, 0.1% Triton X-100, and 10% glycerol at pH 7.4 was used to adjust NaCl concentration. The volume of no-salt buffer (V_{nosalt}) required for each formulation was calculated using the following equation:

$$V_{nosalt}=20\times\frac{500\text{-}C_{NaCl}}{500} \quad (1)$$

where C_{NaCl} represents the desired concentration of NaCl in the solution. To adjust the protein concentration, further dilution with stock buffer was performed. The volume of stock buffer (V_{stock}) required can be calculated as follows:

$$V_{stock}=20\times\left(1\text{-}\frac{500\text{-}C_{NaCl}}{500}\text{-}\frac{C_{protein}}{C_{sprotein}}\right) \quad (2)$$

where $C_{protein}$ denotes the target protein concentration, and $C_{sprotein}$ is the concentration of the protein stock. Subsequently, FIB1-GFP-RIAD stocks were thawed on ice and added to the mixtures of no-salt buffer and stock buffer to achieve a final volume of 20 μL. The resulting mixtures were incubated at room temperature for 30 minutes to allow for phase separation. 5 μL of the sample was applied to glass slides and covered with a 20×20 mm coverslip before being examined using a Zeiss LSM980 confocal microscope equipped with a 63× oil immersion objective (numerical aperture 1.4), laser launches, and an iXon EM-CCD camera. The GFP signal was excited with a 488-nm laser, and the emission was collected in the range of 510-560 nm.

Fluorescent labeling of MenH-RIDD. Fluorescent labeling of MenH-RIDD was achieved using cyanine 5 N-hydroxysuccinimide (Cy5-NHS, Macklin) according to the manufacturer's protocol. Briefly, a ten-fold molar excess of Cy5-NHS, dissolved in dimethyl sulfoxide (DMSO), was added to the purified MenH-RIDD. The resulting mixture was incubated at 4 °C for 2 hours in the dark to facilitate labeling. Excess Cy5 dye was subsequently removed via size exclusion chromatography (SEC) on an AKTA pure system.

All-atom molecular dynamics (MD) simulations. To investigate how conformational dynamics of the FIB1-RIAD/ RIDD structural scaffold affect client enzyme MenH's activity, we performed molecular modeling and all-atom MD simulations. FIB1-GFP-RIAD (the sequence as shown in Supplementary Table 5) and MenH-RIDD dimer structures were constructed using AI-based Chai-1 modeling method [3]. The structure with the best performed pLDDT score was selected for 100 ns MD simulation to refine the modeling structure. Molecular docking of the MenH substrate SEPHCHC targeting the MenH dimer was performed using Autodock Vina (v1.2.7) [4]. The MenH-SEPHCHC reference system was built using the crystal structure of MenH (PDB ID: 4MYD, Chain A, residues 1-252) and the docked pose of SEPHCHC obtained from AutoDock Vina. Moreover, the apo structure of MenH was also taken

as the reference system. Three independent 500 ns MD simulations were performed for each system: the FIB1-GFP-RIAD/MenH-RIDDs/SEPHCHC complex, the MenH-SEPHCHC complex, and the apo-MenH system.

All MD simulations were performed using Amber (v23) with the Amber14SB force field on a Linux-based high-performance computing (HPC) cluster [5]. The protein and ligand structure for system was solvated in a cubic TIP3P water box with 1 nm distance from the edge and was neutralized by 0.15 M sodium ions. System dimensions and atom counts were as follows: (1) FIB1-GFP-RIAD/MenH-RIDDs/SEPHCHC complex: 152 × 92 × 132 $Å^3$ box containing 159,725 atoms; (2) MenH-SEPHCHC complex: 74 × 76 × 67 $Å^3$ box with 30,425 atoms; (3) apo-MenH system: 74 × 76 × 67 $Å^3$ box with 30,401 atoms. The system's temperature gradually heated to 300 K over 100 ps to perform the 10 ns NVT equilibration and 10 ns NPT equilibration, after 4 steps of energy minimization. The production MD simulations at 300 K and 1 atm were carried out with the LINCS algorithm to restrain the hydrogen positions at their equilibrium distances, which allowed the use of an integration time step of 2 fs. Production simulations employed Langevin dynamics as the thermostat and isotropic position scaling as the barostat. Energies and coordinates were saved every 10 ps for the postproduction analysis of the MD simulations. MD trajectories were analyzed with cpptraj for biomolecular processing and PyMOL (v3.1) for visualization.

Confocal fluorescent imaging of living cells. Samples were prepared as follows. A single colony of *E. coli* cells expressing the protein of interest was inoculated into 6 mL LB media supplemented with the proper antibiotics from a plate and incubated overnight at 37°C with shaking at 200 rpm. The overnight culture was then diluted 100-fold in 50 mL LB medium containing the same antibiotic. Proteins expression of was induced by adding 1 mM IPTG (for FIB1-GFP-RIAD or GFP-RIAD) and/or 0.3% (w/v) arabinose (for mCherry , mCherry-RIDD or Rluc-RIDD). At designated time points, 1 mL culture was harvested by centrifugation and washed three times with PBS. The pelleted cells were resuspended in PBS and 1.5 μL of the sample was placed on glass slides pre-treated with 0.3% poly-L-lysine. For experiments involving the supplementation of glycine or tryptophan, 0.2 mM of the amino acid was added to the cell culture following the induction of FIB1-GFP-RIAD. To maintain the concentration of the added amino acids, a specific amount was supplemented to the culture every two hours, based on the consumption rates reported in previous studies[6, 7].

All fluorescence microscopy experiments on living *E. coli* cells were conducted using the Zeiss LSM980 confocal microscope. GFP signal was excited using a 488-nm laser at 0.5% power and collected between 510-560 nm, while mCherry signal was detected through a 594-nm laser at 0.2% power and collected between 600-670 nm. For in-cell FRAP, the procedure mirrored that of in vitro FRAP, except a spot measuring 1 μm in diameter was photobleached at one pole of the cell.

Western blot analysis. *E. coli* cells with or without catalytic condensates, were harvested by centrifugation at indicated time points. The pelleted cells were washed three times with PBS and mixed with loading buffer for SDS-PAGE. After boiling the samples at 95°C for 5 minutes, they were resolved on a 12% SDS-PAGE gel. Proteins transfer from SDS-PAGE gels to nitrocellulose membranes were achieved using a semi-dry transfer unit (Amersham

Biosciences). The nitrocellulose membranes were subsequently blocked with 5% non-fat milk powder in TBST (50 mM Tris, 0.15 M NaCl, and 0.05% (w/v) Tween 20, pH 7.6)) for 1 h at 4°C. Following blocking, the membranes were incubated with a primary antibody (abcam, ab19321, goat anti-S tag, diluted 1:15,000) for 3 h. This was followed by an incubation with a horseradish peroxidase (HRP)-labeled secondary antibody (abcam, ab6885, donkey anti-goat IgG H&L (HRP), diluted 1:2,000) for 1 h. Finally, bands were visualized using Enhanced Chemiluminescence (ECL, Servicebio).

Supplementary References

(1) Li, X.; Romero, P.; Rani, M.; Dunker, A. K.; Obradovic, Z., Predicting Protein Disorder for N-, C- and Internal Regions. *Genome Inform* **1999**, *10*, 30-40.

(2) Dosztányi, Z., Prediction of protein disorder based on IUPred. *Protein Sci.* **2018**, *27* (1), 331-340.

(3) team, C. D.; Boitreaud, J.; Dent, J.; McPartlon, M.; Meier, J.; Reis, V.; Rogozhonikov, A.; Wu, K., Chai-1: Decoding the molecular interactions of life. *BioRxiv* **2024**, *10*. 10.615955.

(4) Trott, O.; Olson, A. J., AutoDock Vina: improving the speed and accuracy of docking with a new scoring function, efficient optimization, and multithreading. *J. Comput. Chem* **2010,** *31* (2), 455-461.

(5) Case, D. A.; Aktulga, H. M.; Belfon, K.; Cerutti, D. S.; Cisneros, G. A.; Cruzeiro, V. W. D.; Forouzesh, N.; Giese, T. J.; Götz, A. W.; Gohlke, H., AmberTools. *J. Chem. Inf. Model* **2023,** *63* (20), 6183-6191.

(6) Koch, A. L., The kinetics of gycine incorporation by Escherichia coli. *J. Biol. Chem.* **1955**, *217* (2), 931-945.

(7) Scott, T. A.; Happold, F. C., Studies on the inhibition of induced tryptophanase synthesis in Escherichia coli by the simultaneous presence of fermentable carbohydrate and aromatic amino acids during growth. *Biochem. J* **1962**, *82* (3), 407-412.

Supplementary Figure 2. SDS-PAGE of purified proteins.

Supplementary Figure 2a

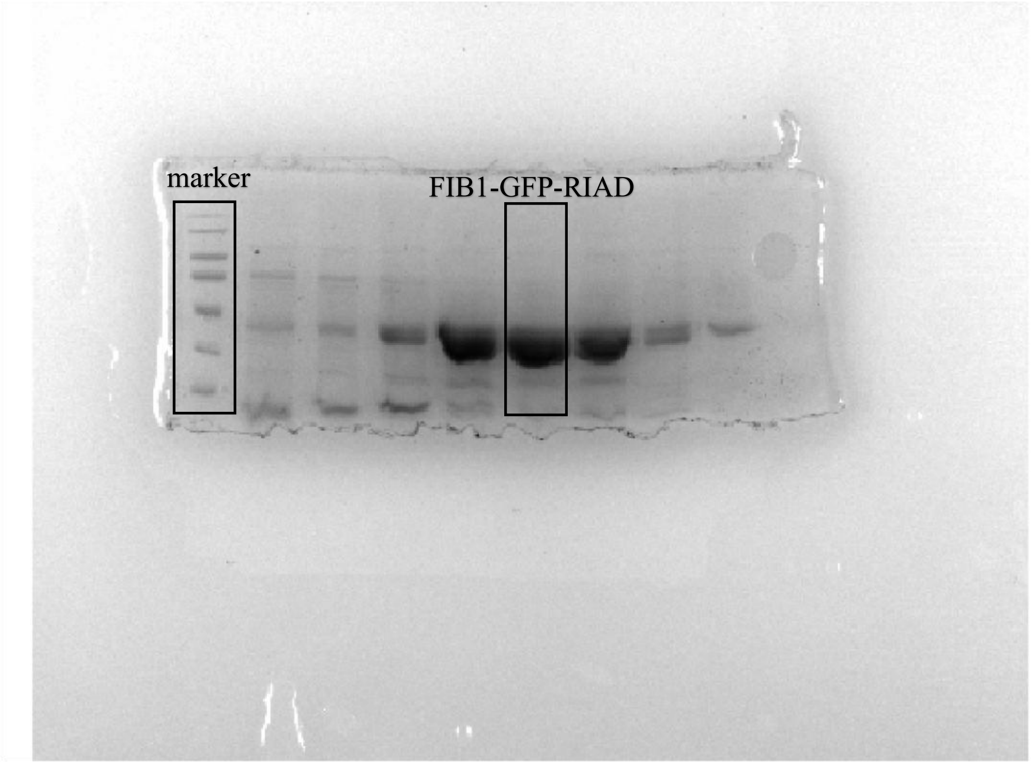

Supplementary Figure 2b

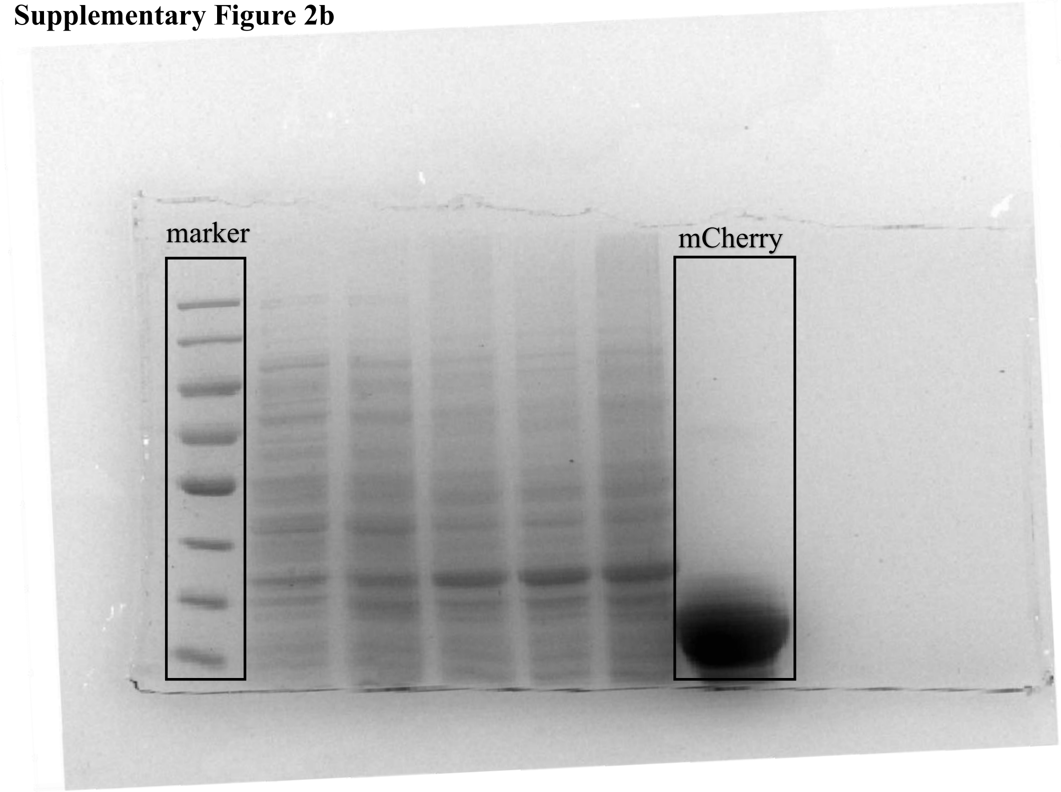

Supplementary Figure 2c

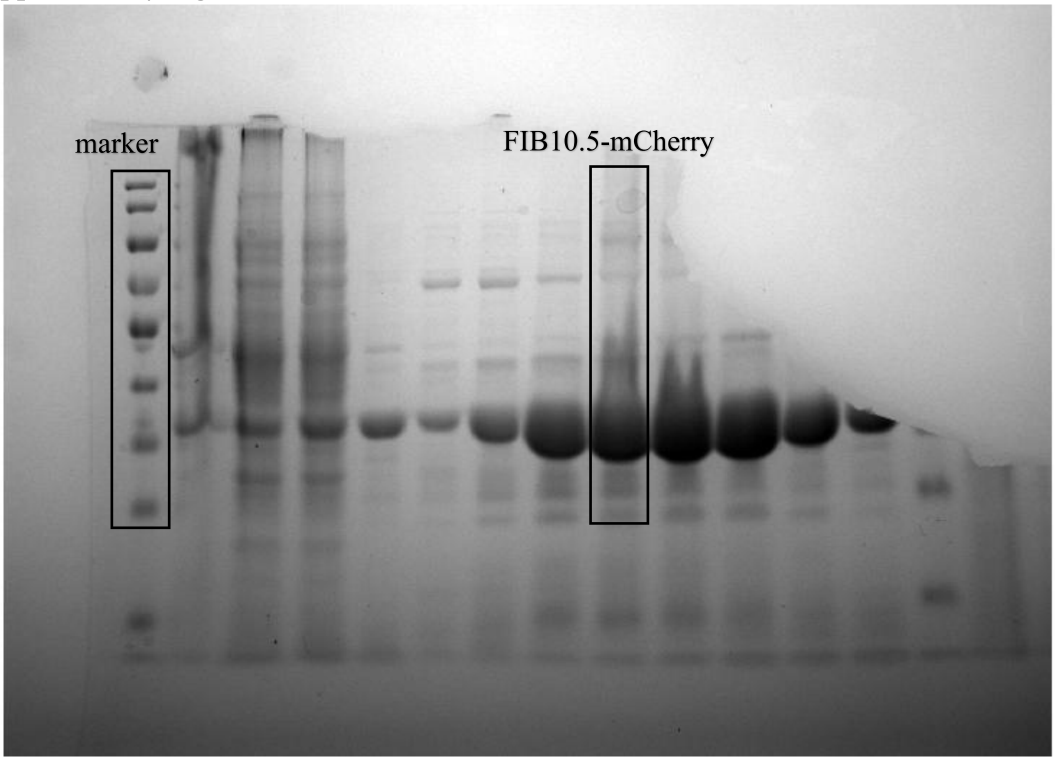

Supplementary Figure 2d

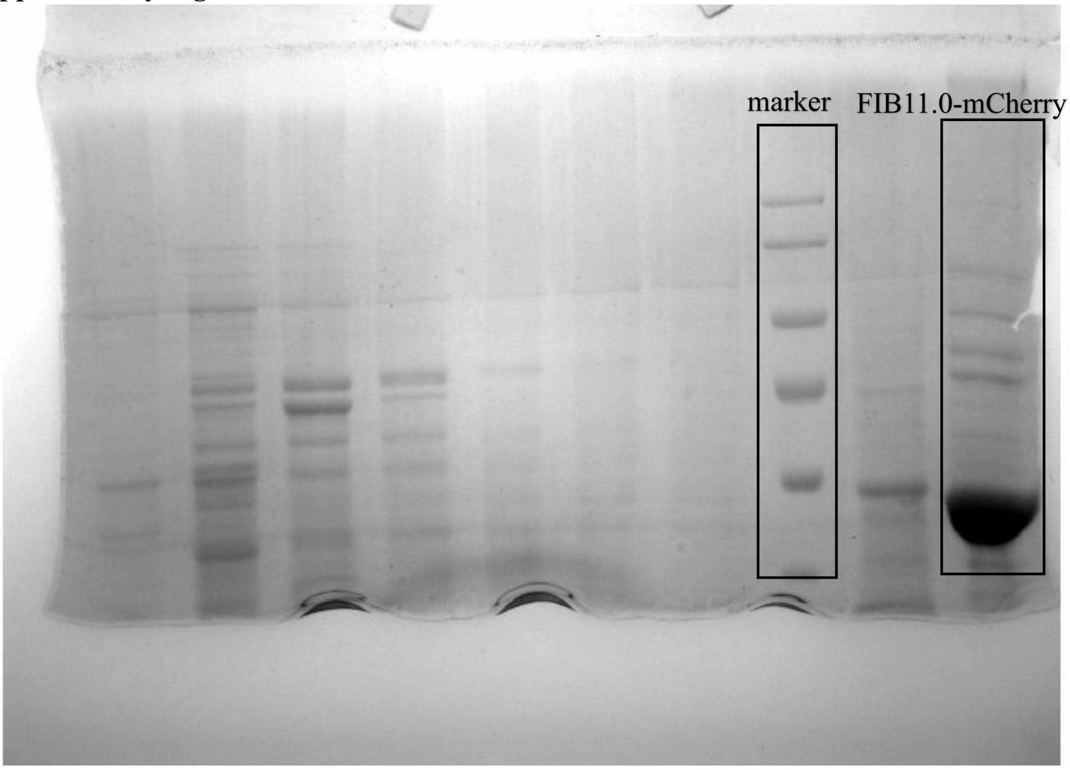

Supplementary Figure 2e

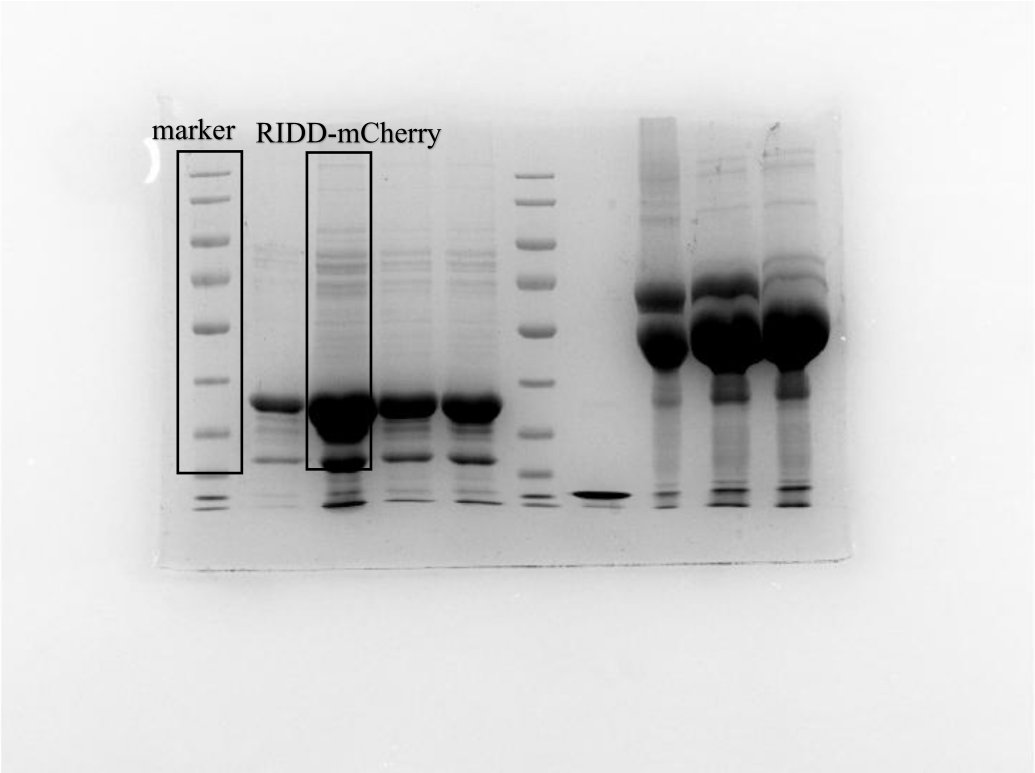

Supplementary Figure 2f

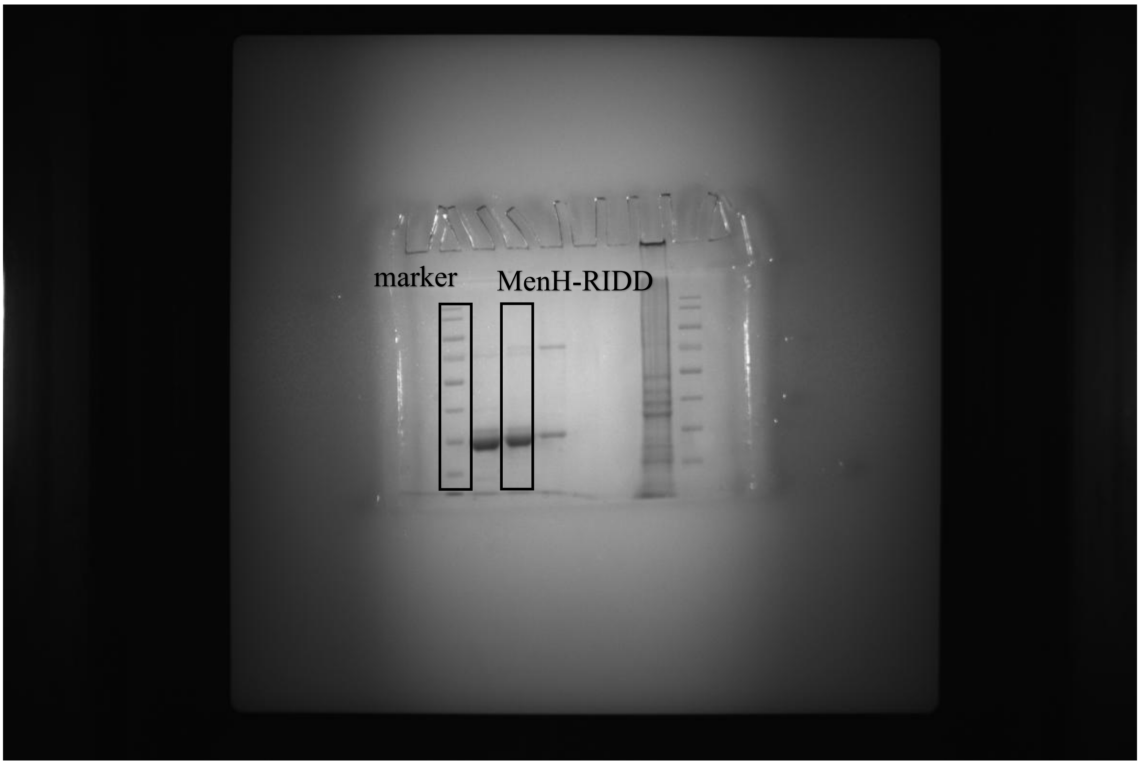

Supplementary Figure 17. Comparison of luciferase expression levels between Erluc0 and Erluc1.

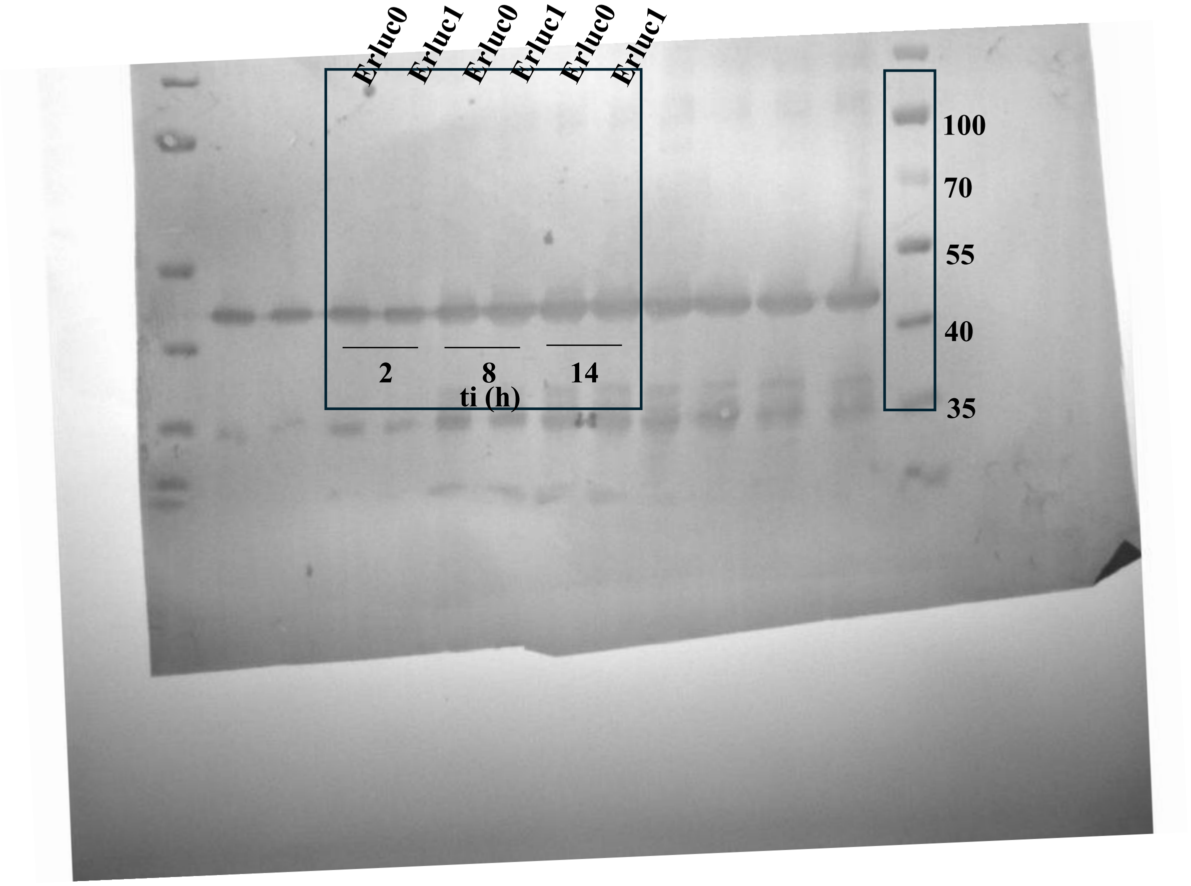

Supplementary Figure 21a. Comparison of luciferase expression levels between Erluc0 and Erluc1 in the presence of 0.2 mM glycine.

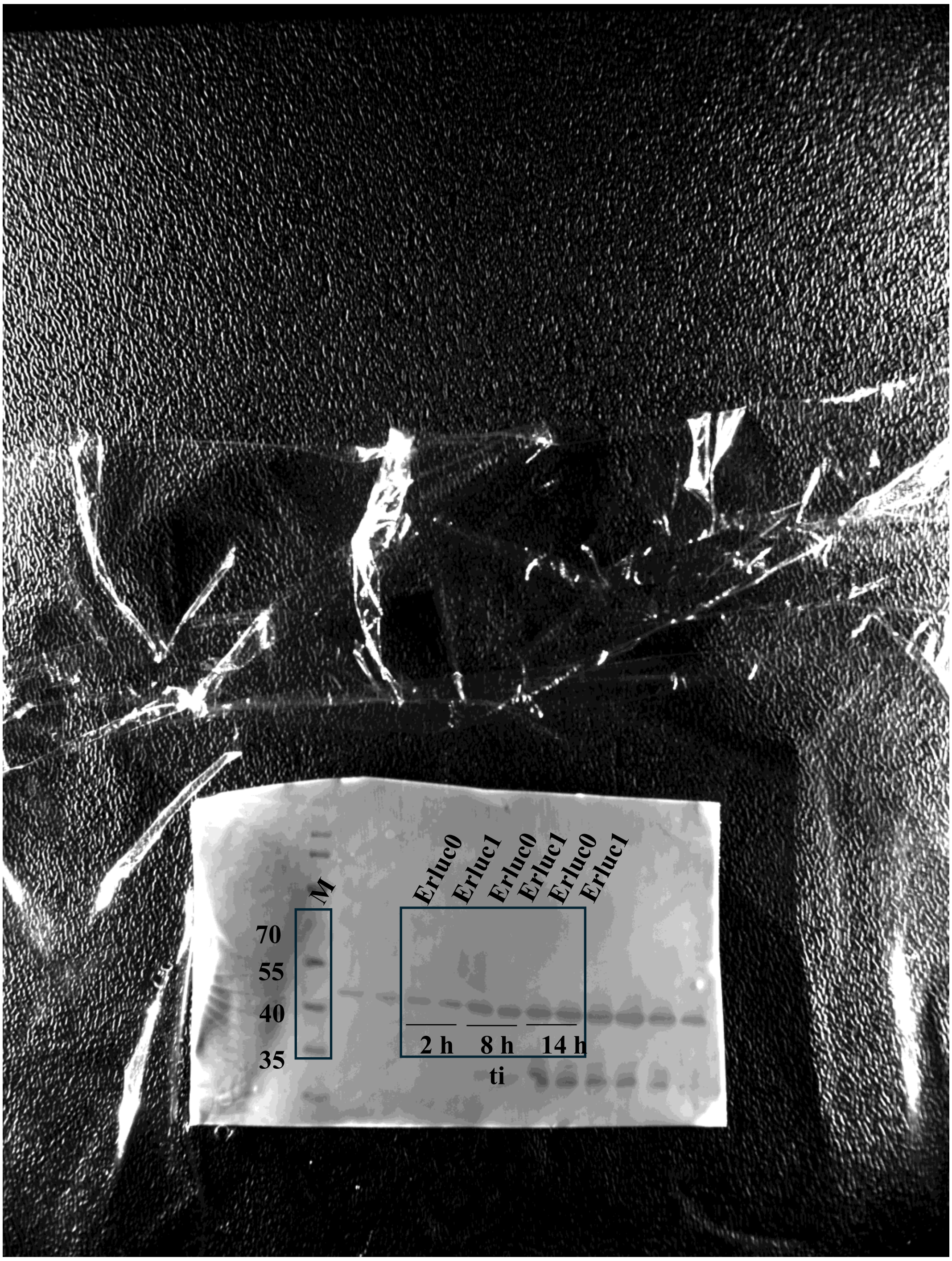

Supplementary Figure 21b. Comparison of luciferase expression levels between Erluc0 and Erluc1 in the presence of 0.2 mM tryptophan.

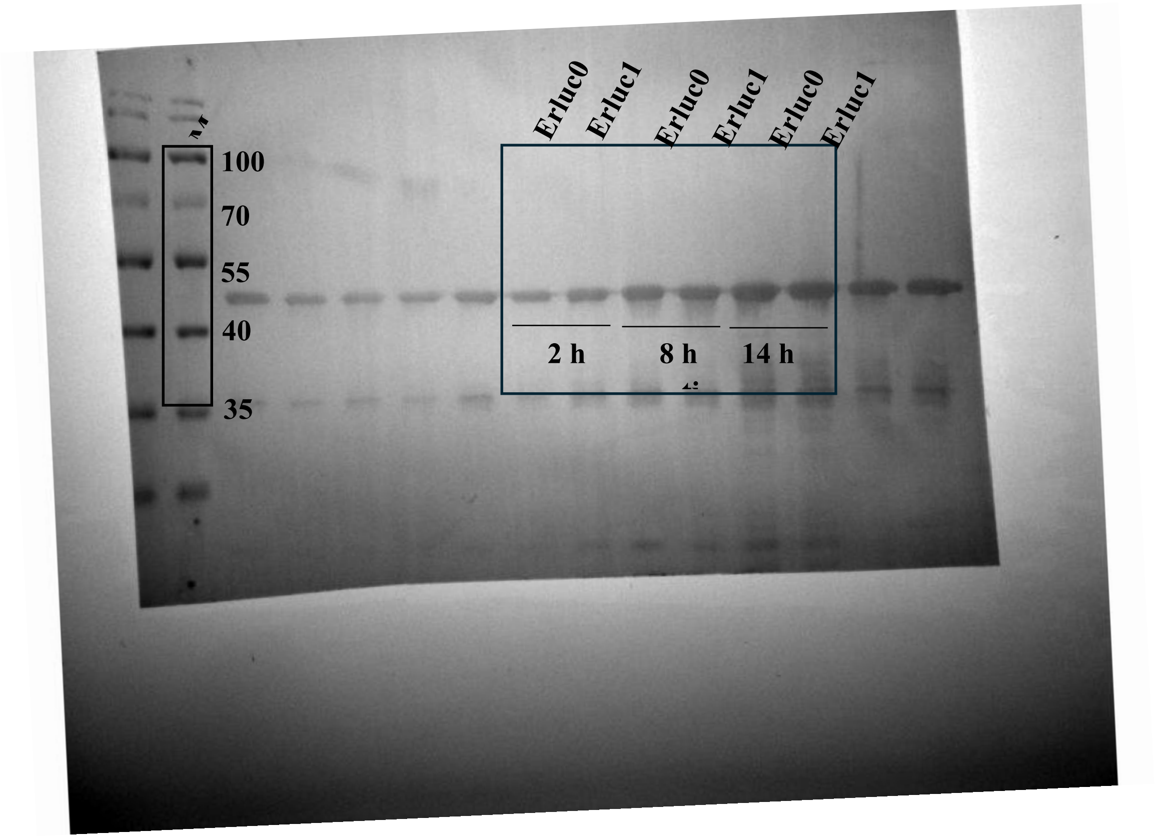

Supplement: Supplementary file 1 — Supplementary Information [file 41467_2025_62074_MOESM1_ESM.pdf]
