# Supplementary material for: Time-dependent catalytic activity in aging condensates
Source: Nat Commun. 2025 Jul 29;16:6959. doi: 10.1038/s41467-025-62074-5 (PMC12307654; doi:10.1038/s41467-025-62074-5)
Supplement: Supplementary file 2 — Description of Additional Supplementary Files [file 41467_2025_62074_MOESM2_ESM.pdf]

Description of Additional Supplementary Files

File Name: Supplementary Movie 1
Description: Three-dimensional reconstructed electron tomography and model reveal the intracellular morphology and spatial distribution of FIB1-GFP-RIAD condensates formed after 2 hours of expression shown in Fig. 5b and 5c.

File Name: Supplementary Movie 2
Description: Three-dimensional reconstructed electron tomography and model reveal the intracellular morphology and spatial distribution of FIB1-GFP-RIAD condensates formed after 8 hours of expression shown in Fig. 5b and 5c.

File Name: Supplementary Movie 3
Description: Three-dimensional reconstructed electron tomography and model reveal the intracellular morphology and spatial distribution of FIB1-GFP-RIAD condensates formed after 14 hours of expression shown in Fig. 5b and 5c.

File Name: Supplementary Data 1
Description: The initial coordinate file for the apo-MenH system was used to generate Supplementary Fig. 12.

File Name: Supplementary Data 2
Description: The final output coordinate file for the apo-MenH system was used to generate Supplementary Fig. 12.

File Name: Supplementary Data 3
Description: The initial coordinate file for the FIB1-GFP-RIAD/MenH-RIDDs/SEPHCHC complex system was used to generate Supplementary Fig. 12.

File Name: Supplementary Data 4
Description: The final output coordinate file for the FIB1-GFP-RIAD/MenH-RIDDs/SEPHCHC complex system was used to generate Supplementary Fig. 12.

File Name: Supplementary Data 5
Description: The initial coordinate file for a MenH-SEPHCHC complex system was used to generate Supplementary Fig. 12.

File Name: Supplementary Data 6
Description: The final output coordinate file for a MenH-SEPHCHC complex system was used to generate Supplementary Fig. 12.
