# Supplementary material for: Time-dependent catalytic activity in aging condensates
Source: Nat Commun. 2025 Jul 29;16:6959. doi: 10.1038/s41467-025-62074-5 (PMC12307654; doi:10.1038/s41467-025-62074-5)
Supplement: Supplementary file 12 — Reporting Summary [file 41467_2025_62074_MOESM12_ESM.pdf]

nature portfolio

Corresponding author(s): Wei Kang: kangwei@dlut.edu.cn
Bo Li: bli10@kennesaw.edu
Chuang Xue: xue.1@dlut.edu.cn
Juncai Ma: juncaima@cuhk.edu.hk

Last updated by author(s): Jun 26, 2025

Reporting Summary

Nature Portfolio wishes to improve the reproducibility of the work that we publish. This form provides structure for consistency and transparency in reporting. For further information on Nature Portfolio policies, see our Editorial Policies and the Editorial Policy Checklist.

Statistics

For all statistical analyses, confirm that the following items are present in the figure legend, table legend, main text, or Methods section.

n/a	Confirmed	
[ ]	[x]	The exact sample size (n) for each experimental group/condition, given as a discrete number and unit of measurement
[ ]	[x]	A statement on whether measurements were taken from distinct samples or whether the same sample was measured repeatedly
[ ]	[x]	The statistical test(s) used AND whether they are one- or two-sided<br>*Only common tests should be described solely by name; describe more complex techniques in the Methods section.*
[x]	[ ]	A description of all covariates tested
[x]	[ ]	A description of any assumptions or corrections, such as tests of normality and adjustment for multiple comparisons
[ ]	[x]	A full description of the statistical parameters including central tendency (e.g. means) or other basic estimates (e.g. regression coefficient) AND variation (e.g. standard deviation) or associated estimates of uncertainty (e.g. confidence intervals)
[ ]	[x]	For null hypothesis testing, the test statistic (e.g. F, t, r) with confidence intervals, effect sizes, degrees of freedom and P value noted<br>*Give P values as exact values whenever suitable.*
[x]	[ ]	For Bayesian analysis, information on the choice of priors and Markov chain Monte Carlo settings
[x]	[ ]	For hierarchical and complex designs, identification of the appropriate level for tests and full reporting of outcomes
[x]	[ ]	Estimates of effect sizes (e.g. Cohen's d, Pearson's r), indicating how they were calculated

Our web collection on statistics for biologists contains articles on many of the points above.

Software and code

Policy information about availability of computer code

Data collection	Confocal imaging and photobleaching: a Zeiss LSM980 confocal fluorescence microscope equipped with an iXon EM-CCD camera and a 63× oil immersion objective (numerical aperture 1.4).<br>Particle tracking microrheology: a Nikon SD-SORA spinning disk confocal microscope outfitted with an sCMOS camera and a 40× water immersion objective (numerical aperture 1.0).<br>Transmission electron microscopy (TEM): a JEM-F200 TEM coupled with a Gatan Rio16 camera.<br>Thin-section TEM: a Hitachi H-7650 TEM furnished with a charge-coupled device (CCD) camera.<br>SDS-PAGE and Western blot: a Bio-Rad gel imaging instrument with a ChemiDoc XRS+ System.
Data analysis	Particle tracking microrheology: MATLAB R2024a v24.1<br>All-atom MD simulations: AmberTools v23 and AutoDock Vina v1.2.7<br>Transmission electron microscopy: Gatan DigitalMicrograph v3.6.1<br>3D electron tomography: IMOD v4.11.25 and QuickTime v7.7.9<br>Further Image analysis: FIJI v1.53f51<br>Curve fitting and statistical analysis: OriginPro 2021 v9.85.204

For manuscripts utilizing custom algorithms or software that are central to the research but not yet described in published literature, software must be made available to editors and reviewers. We strongly encourage code deposition in a community repository (e.g. GitHub). See the Nature Portfolio guidelines for submitting code & software for further information.

Data

Policy information about availability of data

All manuscripts must include a data availability statement. This statement should provide the following information, where applicable:

- Accession codes, unique identifiers, or web links for publicly available datasets
- A description of any restrictions on data availability
- For clinical datasets or third party data, please ensure that the statement adheres to our policy

All data generated or analyzed during this study are included in the manuscript, the supplementary information and the source data. Additional videos are supplied in supplementary videos. Any remaining questions or requests should be addressed to the corresponding author.

Research involving human participants, their data, or biological material

Policy information about studies with human participants or human data. See also policy information about sex, gender (identity/presentation), and sexual orientation and race, ethnicity and racism.

Reporting on sex and gender	No human research participants were used in this study.
Reporting on race, ethnicity, or other socially relevant groupings	No human research participants were used in this study.
Population characteristics	No human research participants were used in this study.
Recruitment	No human research participants were used in this study.
Ethics oversight	No human research participants were used in this study.

Note that full information on the approval of the study protocol must also be provided in the manuscript.

Field-specific reporting

Please select the one below that is the best fit for your research. If you are not sure, read the appropriate sections before making your selection.

☒ Life sciences ☐ Behavioural & social sciences ☐ Ecological, evolutionary & environmental sciences

For a reference copy of the document with all sections, see nature.com/documents/nr-reporting-summary-flat.pdf

Life sciences study design

All studies must disclose on these points even when the disclosure is negative.

Sample size	Sample sizes were not predetermined, because sample sizes were chosen such that significant statistical information (means, standard deviations, and P values) could be derived. We generally used sample sizes of at least three biological replicates, which can provide necessary statistical support. The exact sample sizes (e.g., number of condensates or number of bacteria) are indicated in figure legends.
Data exclusions	No data were excluded from the analysis.
Replication	All data were replicated by at least triple.
Randomization	Allocation was random.
Blinding	Blinding was not relevant to this study, because the analysis was carried out in bacteria.

Reporting for specific materials, systems and methods

We require information from authors about some types of materials, experimental systems and methods used in many studies. Here, indicate whether each material, system or method listed is relevant to your study. If you are not sure if a list item applies to your research, read the appropriate section before selecting a response.

Materials & experimental systems

n/a	Involved in the study	
☐	☒	Antibodies
☒	☐	Eukaryotic cell lines
☒	☐	Palaeontology and archaeology
☒	☐	Animals and other organisms
☒	☐	Clinical data
☒	☐	Dual use research of concern
☒	☐	Plants

Methods

n/a	Involved in the study	
☒	☐	ChIP-seq
☒	☐	Flow cytometry
☒	☐	MRI-based neuroimaging

Antibodies

Antibodies used

Western blot: Goat anti-S tag primary antibody (Cat.No.: ab19321; at 1:15000 dilution) and donkey anti-goat IgG H&L (HRP) secondary antibody (Cat.No.: ab6885; at 1:2000 dilution) were bought from abcam.
CLEM: Rabbit anti-GFP primary antibody was bought from Rockland (Cat.No.: 600-401-215; Lot. No.: 35649; at 1:20 dilution). Alexa Fluor 488 goat anti-rabbit IgG H&L (HRP) secondary antibody was purchased from Invitrogen (Cat.No.: A-11008; Lot. No.: 2622404; at 1:200 dilution).

Validation

Goat anti-S tag primary antibody:
Citation: Lin, D. L.; Inoue, T.; Chen, Y. J.; Chang, A.; Tsai, B.; Tai, A. W., The ER Membrane Protein Complex Promotes Biogenesis of Dengue and Zika Virus Non-structural Multi-pass Transmembrane Proteins to Support Infection. Cell Rep. 2019, 27(6), 1666–1674.
Manufacturer's description: https://www.abcam.cn/products/primary-antibodies/s-tag-antibody-ab19321.html

Donkey anti-goat IgG H&L (HRP) secondary antibody:
Citation: Bilgic, S. N.; Domaniku, A.; Toledo, B.; Agca, S.; Weber, B. Z. C.; Arabaci, D. H.; Ozornek, Z.; Lause, P.; Thissen, J. P.; Loumaye, A.; Kir, S., EDA2R-NIK signaling promotes muscle atrophy linked to cancer cachexia. Nature 2023, 617(7962), 827–834.
Manufacturer's description: https://www.abcam.cn/products/secondary-antibodies/donkey-goat-igg-hl-hrp-ab6885.html

Rabbit anti-GFP primary antibody:
Citation: Chung, C.; Verheijen, B. M.; Zhang, X.; Huang, B.; Coakley, A.; McGann, E.; Wade, E.; Dinep-Schneider, O.; LaGosh, J.; Anagnostou, M. E.; Simpson, S.; Thomas, K.; Ernst, M.; Rattray, A.; Lynch, M.; Kashlev, M.; Benayoun, B. A.; Li, Z.; Strathern, J.; Gout, J. F.; Vermulst, M., The fidelity of transcription in human cells. Proc. Natl. Acad. Sci. U.S.A. 2023, 120(5), e2210038120.
Manufacturer's description: https://www.rockland.com/categories/primary-antibodies/gfp-antibody-600-401-215/

Alexa Fluor 488 goat anti-rabbit IgG H&L (HRP) secondary antibodies:
Citation: Chung, C.; Verheijen, B. M.; Zhang, X.; Huang, B.; Coakley, A.; McGann, E.; Wade, E.; Dinep-Schneider, O.; LaGosh, J.; Anagnostou, M. E.; Simpson, S.; Thomas, K.; Ernst, M.; Rattray, A.; Lynch, M.; Kashlev, M.; Benayoun, B. A.; Li, Z.; Strathern, J.; Gout, J. F.; Vermulst, M., The fidelity of transcription in human cells. Proc. Natl. Acad. Sci. U.S.A. 2023, 120(5), e2210038120.
Manufacturer's description: https://www.thermofisher.cn/cn/zh/antibody/product/Goat-anti-Rabbit-IgG-H-L-Cross-Adsorbed-Secondary-Antibody-Polyclonal/A-11008

Plants

Seed stocks

Report on the source of all seed stocks or other plant material used. If applicable, state the seed stock centre and catalogue number. If plant specimens were collected from the field, describe the collection location, date and sampling procedures.

Novel plant genotypes

Describe the methods by which all novel plant genotypes were produced. This includes those generated by transgenic approaches, gene editing, chemical/radiation-based mutagenesis and hybridization. For transgenic lines, describe the transformation method, the number of independent lines analyzed and the generation upon which experiments were performed. For gene-edited lines, describe the editor used, the endogenous sequence targeted for editing, the targeting guide RNA sequence (if applicable) and how the editor was applied.

Authentication

Describe any authentication procedures for each seed stock used or novel genotype generated. Describe any experiments used to assess the effect of a mutation and, where applicable, how potential secondary effects (e.g. second site T-DNA insertions, mosiacism, off-target gene editing) were examined.
