# Supplementary figures and images for: Time-dependent catalytic activity in aging condensates
Source: Nat Commun. 2025 Jul 29;16:6959. doi: 10.1038/s41467-025-62074-5 (PMC12307654; doi:10.1038/s41467-025-62074-5)

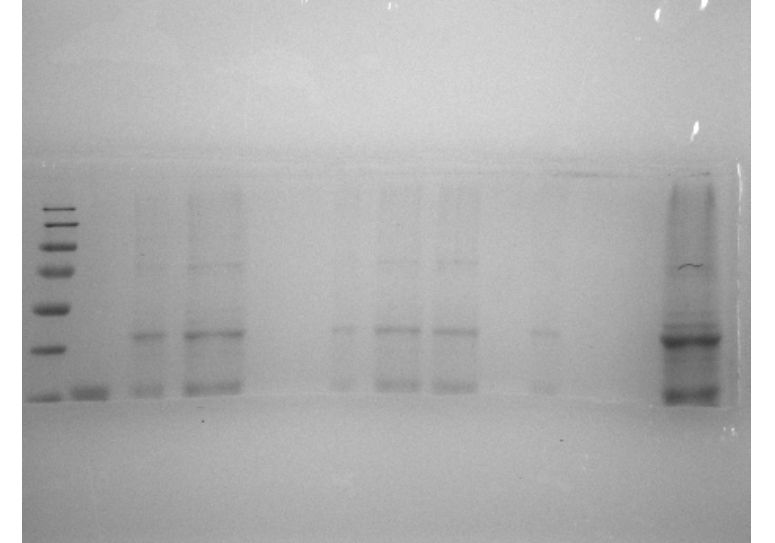

Supplement: Supplementary file 14 — Source Data [file 41467_2025_62074_MOESM14_ESM.zip › Unprocessed_Gel_Fig3d.tif]
